# Basal epithelial tissue folding is mediated by differential regulation of microtubules

**DOI:** 10.1242/dev.167031

**Published:** 2018-11-19

**Authors:** Mike R. Visetsouk, Elizabeth J. Falat, Ryan J. Garde, Jennifer L. Wendlick, Jennifer H. Gutzman

**Affiliations:** Department of Biological Sciences, University of Wisconsin-Milwaukee, Milwaukee, Wisconsin, 53201, USA

**Keywords:** Morphogenesis, Cell shape, Neuroepithelium, Wnt5b, Microtubules, Zebrafish

## Abstract

The folding of epithelial tissues is crucial for development of three-dimensional structure and function. Understanding this process can assist in determining the etiology of developmental disease and engineering of tissues for the future of regenerative medicine. Folding of epithelial tissues towards the apical surface has long been studied, but the molecular mechanisms that mediate epithelial folding towards the basal surface are just emerging. Here, we utilize zebrafish neuroepithelium to identify mechanisms that mediate basal tissue folding to form the highly conserved embryonic midbrain-hindbrain boundary. Live imaging revealed Wnt5b as a mediator of anisotropic epithelial cell shape, both apically and basally. In addition, we uncovered a Wnt5b-mediated mechanism for specific regulation of basal anisotropic cell shape that is microtubule dependent and likely to involve JNK signaling. We propose a model in which a single morphogen can differentially regulate apical versus basal cell shape during tissue morphogenesis.

## Abstract (precis)

**Summary:** Examination of cell shape changes during zebrafish neuroepithelium tissue folding reveals that Wnt5b specifically regulates basal anisotropic cell shape via a microtubule-dependent mechanism, likely involving JNK signaling.

## INTRODUCTION

The folding of epithelial tissues is essential for the generation of complex three-dimensional organ structures during morphogenesis. Localized and coordinated cell shape changes drive these morphogenetic processes via signal transduction events that lead to changes in cytoskeletal activity (Munjal and Lecuit, 2014). The pseudostratified neuroepithelium that gives rise to the central nervous system has gained significant attention for its complex structure and the substantial shape changes that occur during its development (Norden, 2017). Apical constriction mediates epithelial folding towards the apical surface during neurulation, and many of the molecular mechanisms that mediate apical constriction have been elucidated in both vertebrate and invertebrate systems (Hunter and Fernandez-Gonzalez, 2017; Martin and Goldstein, 2014). Later in embryonic brain morphogenesis, the neuroepithelium folds towards the basal surface to generate the highly conserved midbrain-hindbrain boundary (MHB). However, a significant gap remains in our understanding of the molecular mechanisms that mediate basal tissue folding.

Basal constriction was first clearly defined as an essential cell shape change required for basal epithelial folding during formation of the MHB in zebrafish (Gutzman et al., 2008). Since this finding, basal constriction has been described during zebrafish optic cup morphogenesis, *Ciona* notochord cell elongation, *Drosophila* egg chamber elongation, and *Hydra* bud formation (Bogdanović et al., 2012; Dong et al., 2011; He et al., 2010; Holz et al., 2017; Martinez-Morales et al., 2009; Sidhaye and Norden, 2017). These results, across both vertebrate and invertebrate systems, suggest that basal constriction is widespread and required for diverse morphogenetic events during development. However, the molecular mechanisms that mediate basal constriction and the cell shape changes required for basal epithelial folding are only just emerging.

Common signaling molecules and cytoskeletal components have been demonstrated to mediate tissue folding, both apically and basally. Oscillating contractions of the actomyosin network, localized apically, mediate apical constriction during ventral furrow formation in *Drosophila* (Martin et al., 2009; Vasquez et al., 2014). Similarly, basally localized actomyosin-mediated contractions have been shown to regulate *Drosophila* egg chamber elongation and invagination of the retinal neuroepithelium (He et al., 2010; Nicolas-Perez et al., 2016; Sidhaye and Norden, 2017). During MHB formation, actin accumulates basally at the point of deepest constriction and the non-muscle myosin II (NMII) proteins NMIIA and NMIIB differentially mediate cell shape changes that are required for the basal fold (Gutzman et al., 2008, 2015). Calcium also has a role in mediating apical constriction during neural tube closure (Christodoulou and Skourides, 2015; Suzuki et al., 2017) and functions as an upstream regulator of the basal MHB tissue fold in zebrafish and of basal constriction of the *Drosophila* egg chamber (He et al., 2010; Sahu et al., 2017). In addition, Wnt signaling is important for both apical and basal constriction. During *Xenopus* and *Caenorhabditis elegans* gastrulation, and in shaping mammalian lung epithelium, Wnts mediate apical constriction (Choi and Sokol, 2009; Fumoto et al., 2017; Lee et al., 2006) and Wnt5b is required for basal constriction during MHB morphogenesis (Gutzman et al., 2018).

Although there are several common molecules that regulate both apical and basal epithelial tissue folding, there are also clear distinctions. Apical constriction depends on proper localization of apical complexes including N-cadherin (Cadherin 2), Shroom3 and Celsr1 to coordinate apical actomyosin dynamics during neural tube closure and lens placode invagination (Morita et al., 2010; Nishimura et al., 2012; Plageman et al., 2010). Basal constriction requires basal adhesion molecules such as focal adhesion kinase and β-integrins (Bogdanović et al., 2012; Gutzman et al., 2018), and requires laminin, a crucial component of the basement membrane (Bryan et al., 2016; Gutzman et al., 2008; Nicolas-Perez et al., 2016). However, the molecular mechanisms that mediate basal constriction and basal tissue folding remain unknown.

Here, we utilized the zebrafish MHB, the highly conserved first fold in the vertebrate neuroepithelium (Gibbs et al., 2017), as a morphogenetic model to identify molecular mechanisms that mediate basal tissue folding. We developed a method to measure how these pseudostratified neuroepithelial cells change shape in three dimensions, which led to the identification of anisotropic cell shape changes as the tissue folds. We demonstrate that Wnt5b plays an early role in the regulation of both apical and basal anisotropic cell shape and we determined that Wnt5b differentially and specifically mediates basal anisotropic cell shape through the regulation of microtubules. Our data also suggest that Wnt5b regulation of basal anisotropic cell shape is likely to be mediated through Jun N-terminal kinase (JNK) signaling. We propose a model in which a single morphogen, Wnt5b, is capable of differentially regulating apical and basal cell shape during basal tissue folding. Elucidating the molecular mechanisms that regulate multi-dimensional cell and tissue shape will provide a necessary foundation for determining how different genetic or extrinsic environmental factors may affect morphogenetic processes. These studies will also be important for the future of sculpting organs (Hughes et al., 2018). Engineering tissues with rich *in vivo* architectures could be useful for regenerative medicine, *in vitro* modeling of diseases, and tissue-scale toxicological studies.

## RESULTS

### Three-dimensional neuroepithelial cell shape analysis reveals anisotropic cell shape

**Fig. 1.**
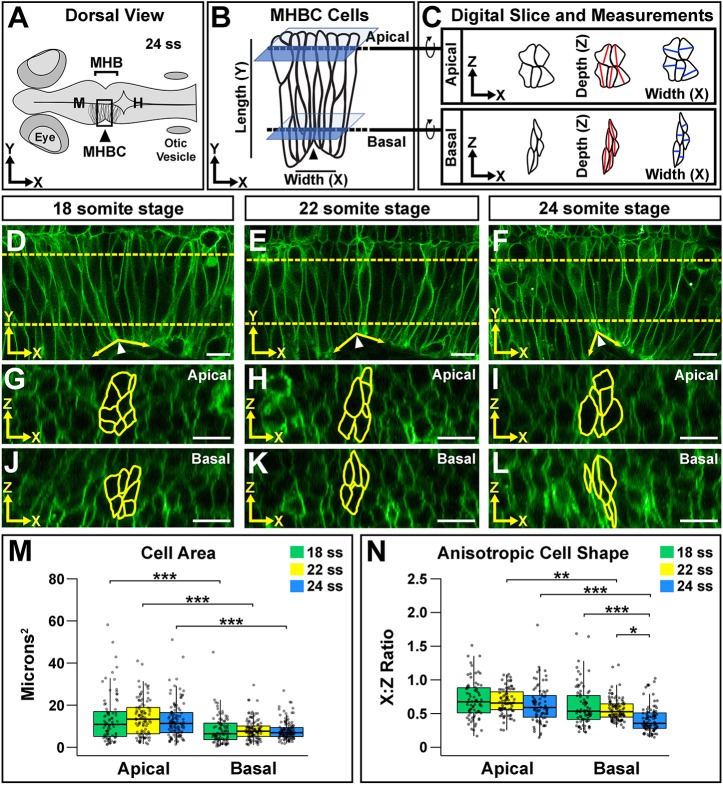
**Neuroepithelial cell shape analysis reveals anisotropic shape that is enhanced basally during morphogenesis.** (A) Diagram of a 24 ss zebrafish embryo. H, hindbrain; M, midbrain. (B) Close-up view of the boxed MHBC region in A showing cell orientation with length (apical-basal, *y*-axis) and width (anterior-posterior, *x*-axis) indicated. Dashed lines indicate where apical and basal digital orthogonal slices were generated for analyses. (C) Digital slices were rotated 90° to the right to reveal apical and basal cell shape in the *xz* plane. Cell area was measured by outlining cells. Cell depth was measured at the deepest point of the cell within 45° of the *z*-axis (red lines). Cell width was measured at the widest point of the cell along the *x*-axis, perpendicular to the depth measurement (blue lines). See also Fig. S1. (D-L) Live confocal images of wild-type embryos injected with memCherry mRNA at 18 ss (D,G,J), 22 ss (E,H,K) and 24 ss (F,I,L). Apical (G-I) and basal (J-L) digital slices, taken at the locations marked by the dashed lines in D-F at the indicated time points, are shown. MHBC cells are outlined in yellow. (M) Quantification of apical and basal cell area. (N) Quantification of anisotropic cell shape using the width (*x*) to depth (*z*) ratio (*x*:*z* ratio). Boxplots indicate the 25th and 75th percentiles and the median. Three independent experiments are represented. 18 ss, *n*=6; 22 ss, *n*=6; 24 ss, *n*=6. *P<0.05, **P<0.01, ***P<0.005. Arrowheads indicate MHBC and arrows indicate MHB tissue angle. Anterior is to the left in all images. Scale bars: 10 μm.

To begin to identify the cellular and molecular mechanisms that mediate basal tissue folding, we used the developing zebrafish MHB as a model. We examined the deepest point of the MHB fold, termed as the midbrain-hindbrain boundary constriction (MHBC) (Fig. 1A) (Gutzman et al., 2008). The cells at the MHBC are part of a single layer of pseudostratified neuroepithelium with apical-basal polarity (Fig. 1B). The brain ventricles develop along the apical cell surface and the basement membrane lines the basal cell surface. We have characterized the cell shape changes that form the MHB by measuring cell length (apical-basal, *y*-axis) and cell width (anterior-posterior, *x*-axis) (Fig. 1B) (Gutzman et al., 2015). To fold the neuroepithelium, cells at the deepest point of the constriction decrease in length relative to cells outside of the MHBC and cells decrease in width throughout the MHB region (Gutzman et al., 2015). These cell shape changes are crucial to the formation of the basal tissue fold, as failure of either one of these cell shape changes results in an overall tissue shape defect. The defect is the formation of an obtuse MHB tissue angle, which looks grossly similar even though the specific cell shape abnormalities can be different (Gutzman et al., 2015). Here, we developed a method to analyze and quantify cell shape in the third dimension, cell depth (dorsal-ventral, *z*-axis), and investigate its role in regulating basal tissue folding (Fig. 1C).

Cells were visualized by injecting one-cell-stage embryos with membrane Cherry (memCherry) or membrane GFP (memGFP) mRNA followed by live confocal microscopy of the MHB neuroepithelium during the early stages of MHB formation, between 18 and 24 somite stage (ss) (Fig. 1D-L). Using *z*-series data, digital orthogonal slices were generated from the apical and basal sides of the MHBC cells (Fig. 1G-L; Fig. S1). From these images, we observed that wild-type MHBC cells had different properties in different dimensions. MHBC cells appeared to have greater depth in the *z*-dimension and less width in the *x*-dimension, revealing that these cells exhibit anisotropic cell shape in the *xz* plane.

### Basal anisotropic cell shape is significantly enhanced between 18 ss and 24 ss

In order to quantify these morphological differences in the *xz* plane, we took three different measurements of the MHBC cells (Fig. 1C; Fig. S1D′,D″,E′,E″). First, we measured cell area in the *xz* plane by manually outlining cells at the MHBC from both apical and basal digital slices (Fig. 1G-L, yellow outlines). Second, we measured the maximal depth of cells in the *z*-dimension, within 45° of the *z*-axis (demonstrated by red lines in Fig. 1C; Fig. S1D″,E″). Finally, we measured the maximal width of the cells in the *x*-dimension, perpendicular to the *z* measurement (demonstrated by blue lines in Fig. 1C; Fig. S1D″,E″). Then, we quantified the anisotropic shape of the cells in the *xz* plane using an *x*:*z* ratio, dividing the width (*x*-dimension) by the depth (*z*-dimension). Anisotropic cell shape is defined as an *x*:*z* ratio greater than or less than one.

Using this quantification method, we examined apical and basal cell area and *x*:*z* ratios of MHBC cells at 18 ss, 22 ss and 24 ss (Fig. 1G-L). MHBC cells had a significantly greater area on the apical side compared with the basal side at each time point examined (Fig. 1M). However, when we compared apical cell area over time or basal cell area over time, no significant differences were detected. *x*:*z* ratio measurements were <1, both apically and basally, which confirmed that the MHBC cells are anisotropic (Fig. 1N). This is consistent with MHBC cells being described as ‘wedge-shaped’ (Gutzman et al., 2018). When we compared only apical *x*:*z* ratios, no significant differences were found (Fig. 1G-I,N), suggesting that the apical anisotropic cell shape does not change during this developmental window. Interestingly, comparison of *x*:*z* ratios among basal digital slices revealed significant changes at 24 ss (Fig. 1J-L,N). Together, these data demonstrate that during the early stages of MHB morphogenesis, MHBC cells have apical and basal polarized cell shape and anisotropic cell shape in the *xz* plane. In general, cells are narrower along the *x*-axis and deeper along the *z*-axis. As cell area in the *xz* plane did not change at these time points, our data suggest that the enhancement of basal anisotropic cell shape contributes to early MHB formation. These cell shape quantification methods, and wild-type characterization, provide a platform for elucidating the molecular mechanisms that mediate basal tissue folding.

### Wnt5b is required for anisotropic cell shape both apically and basally at the MHBC

**Fig. 2.**
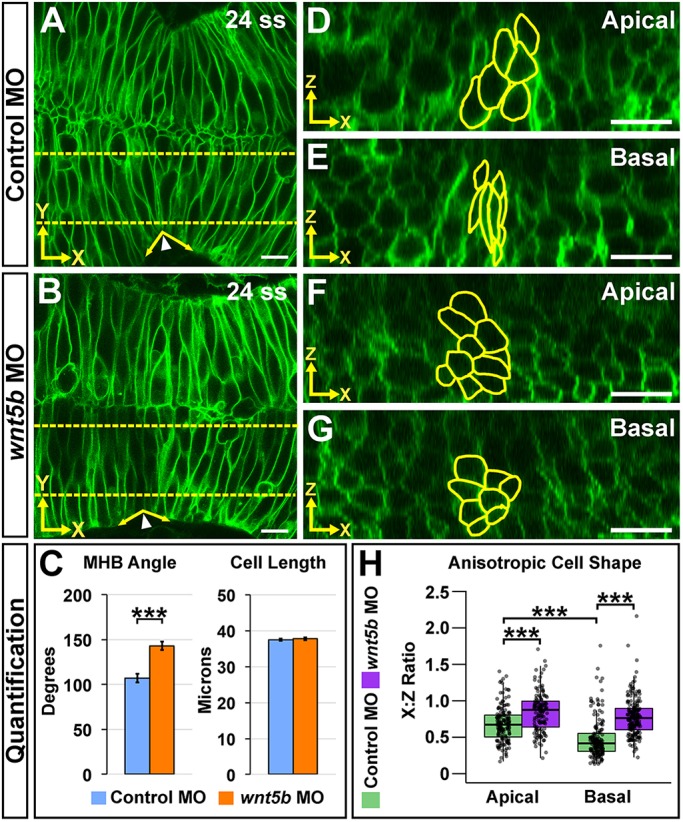
**Wnt5b is required for anisotropic cell shape both apically and basally at the MHBC.** (A,B) Live confocal images of 24 ss wild-type embryos co-injected with memGFP and control MO (A) or *wnt5b* MO (B). (C) Quantification of MHB tissue angle and MHBC cell length (*y*-axis). Data are represented as mean±s.e.m. from eight independent experiments. (D,E) Representative apical (D) and basal (E) digital slices of control morphants. (F,G) Representative apical (F) and basal (G) digital slices of *wnt5b* morphants. (H) Quantification of anisotropic cell shape using *x*:*z* ratio. MHBC cells are outlined in yellow. Boxplots indicate the 25th and 75th percentiles and the median. Eight independent experiments are represented. Control MO, *n*=10; *wnt5b* MO, *n*=10. ***P<0.005. Arrowheads indicate MHBC and arrows indicate MHB tissue angle. Scale bars: 10 μm.

To test for upstream mediators of MHBC cell shape, we previously hypothesized that signaling molecules would be specifically expressed in the MHB region during morphogenesis (Gutzman et al., 2018; Sahu et al., 2017). *wnt5b*, a known morphogen, is expressed at the MHB at the onset of tissue folding (18 ss) (Duncan et al., 2015; Gutzman et al., 2018; Thisse and Thisse, 2005) and has been demonstrated to have a role in basal constriction of MHBC cells late in MHB morphogenesis, at prim-6 (Gutzman et al., 2018). Given the anisotropic cell shape changes we identified between 18 to 24 ss, we further hypothesized that Wnt5b could play an earlier role in MHB formation. In order to test this hypothesis, we utilized live-cell imaging of *wnt5b* antisense oligonucleotide morpholino (MO) knockdown embryos (morphants) (Fig. 2) (De Rienzo et al., 2012; Freisinger et al., 2010; Gutzman et al., 2018; Lin et al., 2010; Young et al., 2014) and *wnt5b pipetail^(ti265)^* mutants (Fig. S2) (Hammerschmidt et al., 1996). Multiple publications confirm the ability of *wnt5b* MOs to clearly phenocopy *pipetail* mutants (Cirone et al., 2008; Lele et al., 2001; Robu et al., 2007); this is also validated here (Fig. S2).

Wild-type embryos were co-injected with memGFP mRNA and either control MO or *wnt5b* MO and imaged at 24 ss. We found that by 24 ss *wnt5b* knockdown perturbed the MHB tissue fold, represented by the MHB angle measurements, but did not affect MHBC cell length (Fig. 2A-C), suggesting that Wnt5b mediates cell shape in a dimension other than the *y*-axis to affect overall tissue shape. From examination of digital slices in the *xz* plane, we found that *wnt5b* knockdown did not affect apical or basal cell area (Fig. S3A). However, quantification of *x*:*z* ratios revealed that knockdown of *wnt5b* perturbed cell depth in the *z*-dimension and cell width in the *x*-dimension, demonstrating that Wnt5b was required to regulate anisotropic cell shape, both apically and basally (Fig. 2D-H). Similar anisotropic cell shape defects were demonstrated using *pipetail^(ti265)^* mutants (Fig. S2E-K); therefore, subsequent experiments were performed using the *wnt5b* MO. We also found no change in the percentage of PH3-positive cells with *wnt5b* knockdown (Fig. S3B-D), suggesting that cell proliferation does not play a role in Wnt5b mediation of MHB morphogenesis. In order to confirm that the abnormal tissue shape observed in *wnt5b* morphants was due to cell shape defects specifically at the MHBC, we measured *x*:*z* ratios in cells 40 μm outside and posterior to the MHBC (Fig. S3E-K). Differences in either apical or basal anisotropic cell shape were not observed between controls and *wnt5b* morphants, demonstrating that *wnt5b*-dependent cell shape changes are region specific (Fig. S3G-K). Together, these data indicate that Wnt5b is required in the early stages of MHB morphogenesis to promote proper MHB tissue folding via regulation of apical and basal anisotropic MHBC cell shape in the *xz* plane.

### Wnt5b regulates tubulin during MHB morphogenesis

**Fig. 3.**
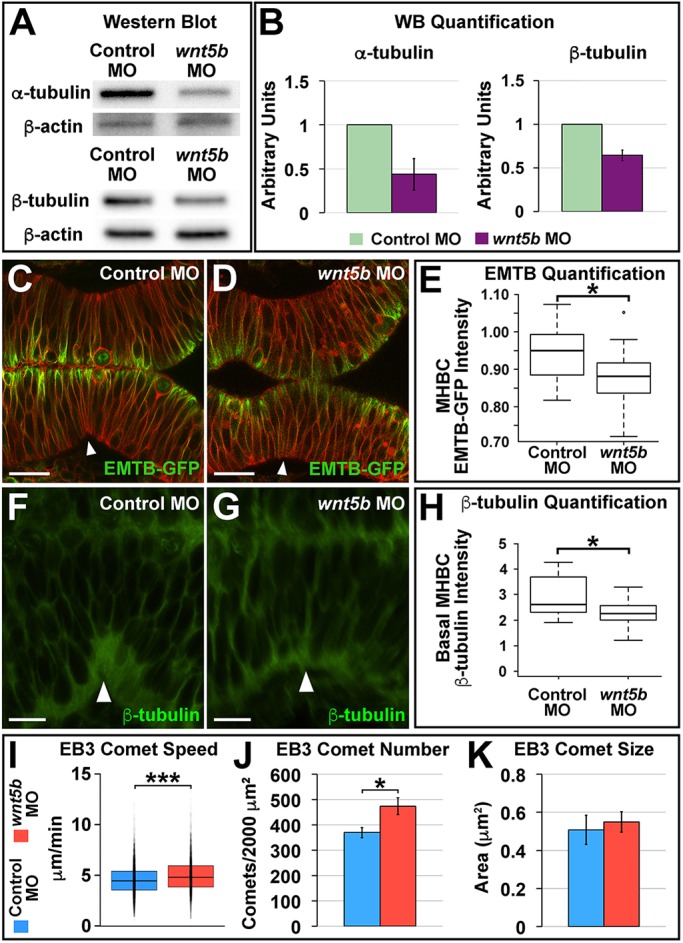
**Wnt5b regulates tubulin protein levels and microtubule dynamics at the MHB.** (A) Representative western blot for α-tubulin and β-tubulin, with β-actin as the control, comparing control MO- and *wnt5b* MO-injected embryos. Head tissue protein was analyzed. (B) Quantification of at least three independent western blot (WB) experiments; α-tubulin and β-tubulin levels were normalized to β-actin. Data are represented as mean±s.d. (C,D) Representative live confocal images showing 10 μm average intensity projections at 22-24 ss of wild-type embryos co-injected with EMTB-GFP, memCherry, and control MO (C) or *wnt5b* MO (D). (E) Quantification of the normalized MHBC EMTB-GFP intensity in control versus *wnt5b* morphants (Fig. S4A-C). Boxplots indicate the 25th and 75th percentiles and the median. Three independent experiments are represented. Control MO, *n*=10; *wnt5b* MO, *n*=7. Scale bars: 20 μm. (F,G) Representative confocal images of β-tubulin immunostaining showing 10 μm average intensity projections at 24 ss of wild-type embryos injected with either control MO (F) or *wnt5b* MO (G). (H) Quantification of the normalized basal MHBC intensity in control versus *wnt5b* morphants. Basal MHBC intensity was divided by the intensity in the middle of the cell (see Fig. S4D-F). Boxplots indicate the 25th and 75th percentiles and the median. Three independent experiments are represented. Control MO, *n*=7; *wnt5b* MO, *n*=6. Scale bars: 10 μm. (I-K) Quantification of control versus *wnt5b* morphants for EB3-GFP comet speed (I), EB3-GFP comet number (J) and EB3-GFP comet size (K). See also Movies 1-4. Boxplots indicate the 25th and 75th percentiles and the median. Three independent experiments are represented. In J,K, data are represented as mean±s.e.m. EB3-GFP comet speed: control MO, *n*=11; *wnt5b* MO, *n*=7. EB3-GFP comet number and comet size: control MO, *n*=11; *wnt5b* MO, *n*=11. *P<0.05, ***P<0.005. Arrowheads indicate MHBC.

Next, we aimed to identify downstream targets of Wnt5b that mediate MHBC anisotropic cell shape. We hypothesized that potential targets would be regulated at the protein level owing to the short time-span between the onset of *wnt5b* expression and MHB morphogenesis. Taking a global approach, we microdissected MHB-specific tissue from control and *wnt5b* knockdown embryos at 22-24 ss using our established method (Sahu et al., 2017), and compared protein populations by two-dimensional (2D) SDS-PAGE. Using mass spectrometry, we identified α-tubulin and β-tubulin as differentially expressed proteins (data not shown). We confirmed our 2D gel analysis by western blot and demonstrated that both α-tubulin and β-tubulin were reduced by approximately 50% in *wnt5b* morphants compared with controls (Fig. 3A,B).

α-Tubulin and β-tubulin are the monomeric building blocks for the microtubule cytoskeleton and both are required for microtubule filament assembly (Etienne-Manneville, 2010). Therefore, we hypothesized that *wnt5b* knockdown, and the observed decrease of α-tubulin and β-tubulin at the MHB, would affect the microtubules within the MHBC cells. We investigated the microtubule filament population *in vivo* with a widely used marker, EMTB-GFP, a chimeric GFP-tagged protein containing the N-terminal microtubule binding domain of ensconsin, a known microtubule-associated protein (MAP) (Bulinski et al., 1999; Norden et al., 2009). Wild-type embryos were co-injected with memCherry and EMTB-GFP mRNA and either control MO or *wnt5b* MO and live imaged at 24 ss. To compare EMTB levels in cells at the MHBC between control and *wnt5b* morphants, MHBC-specific EMTB-GFP intensity was normalized within each embryo by dividing the average EMTB intensity of the MHBC region by the average EMTB intensity of the adjacent midbrain and hindbrain regions (Fig. 3C-E; Fig. S4A-C). We found that the normalized MHBC EMTB-GFP intensity was significantly decreased in *wnt5b* morphants compared with controls (Fig. 3E), suggesting that *wnt5b* knockdown leads to decreased microtubule filaments within the MHBC. Next, we used β-tubulin immunohistochemistry to examine and quantify the basal specific population of microtubules within the MHBC cells. We compared basal and apical β-tubulin levels in controls and *wnt5b* morphants within each embryo by dividing β-tubulin intensity in the basal or apical MHBC region by the β-tubulin intensity in the middle MHBC region (Fig. S4D-F). We found a significant decrease in β-tubulin levels in the basal domain of MHBC cells in *wnt5b* morphants compared with controls (Fig. 3F-H), demonstrating that Wnt5b is essential for the basal microtubule population at the MHBC. There were no differences in apical β-tubulin intensity (Fig. S4G).

From these data, we uncovered an important role for Wnt5b in mediating the microtubule cytoskeleton during MHB basal tissue folding. However, it is known that Wnt signals mediate actomyosin dynamics in other morphogenetic contexts such as gastrulation, heart tube remodeling and lung epithelial morphogenesis (Fumoto et al., 2017; Kim and Davidson, 2011; Lee et al., 2006; Merks et al., 2018). In addition, our previous work revealed a crucial role for non-muscle myosins in mediating the cell shape changes required for MHB basal tissue folding (Gutzman et al., 2015). Therefore, we investigated a potential role for *wnt5b* in mediating actomyosin at the MHBC by examining *in vivo* localization of myosin using GFP-tagged myosin regulatory light chain (MRLC-GFP) (Norden et al., 2009) and actin using phalloidin staining. Following quantification of apical and basal MRLC-GFP and actin localization, no differences were detected at the MHBC when comparing controls with *wnt5b* knockdowns (Fig. S5), suggesting that Wnt5b does not act directly through the actomyosin network to mediate MHB basal tissue folding.

Together, these data demonstrate a role for *wnt5b* in the regulation of tubulin levels and suggest that Wnt5b is important for regulating the microtubule cytoskeleton within the MHBC during early MHB morphogenesis.

### Wnt5b regulates microtubule dynamics during early MHB morphogenesis

As we determined that *wnt5b* mediates α-tubulin, β-tubulin and microtubule filament levels in the MHBC region and basally at the MHBC, we hypothesized that Wnt5b would also be required to regulate microtubule dynamics during early MHB morphogenesis. In order to test this hypothesis, we analyzed microtubule plus-end dynamics *in vivo* by following the fast-growing plus-end microtubule-binding protein EB3, tagged with GFP (Norden et al., 2009). We co-injected memCherry and EB3-GFP mRNA with control MO or *wnt5b* MO and performed time-lapse imaging of the MHB region for 10 min at 24 ss (Movies 1-4). We quantified EB3-GFP microtubule comet speed, comet number and comet size using Fiji (Fig. 3I-K). The EB3-GFP dynamic microtubule quantification was focused below the apical mitotic organizing center, in the more basal domain. We found that EB3-GFP comets moved significantly faster in *wnt5b* morphants compared with controls, at an average of 5 μm per minute compared with 4.5 μm per minute, respectively (Fig. 3I). The number of EB3-GFP comets was also significantly higher in *wnt5b* morphants relative to control morphants (Fig. 3J); however, there was no difference in comet size (Fig. 3K). These data revealed that *wnt5b* is required for regulation of microtubule dynamics within the MHBC.

### Microtubule polymerization is required for basal, but not apical, anisotropic cell shape in MHBC cells at 24 ss

**Fig. 4.**
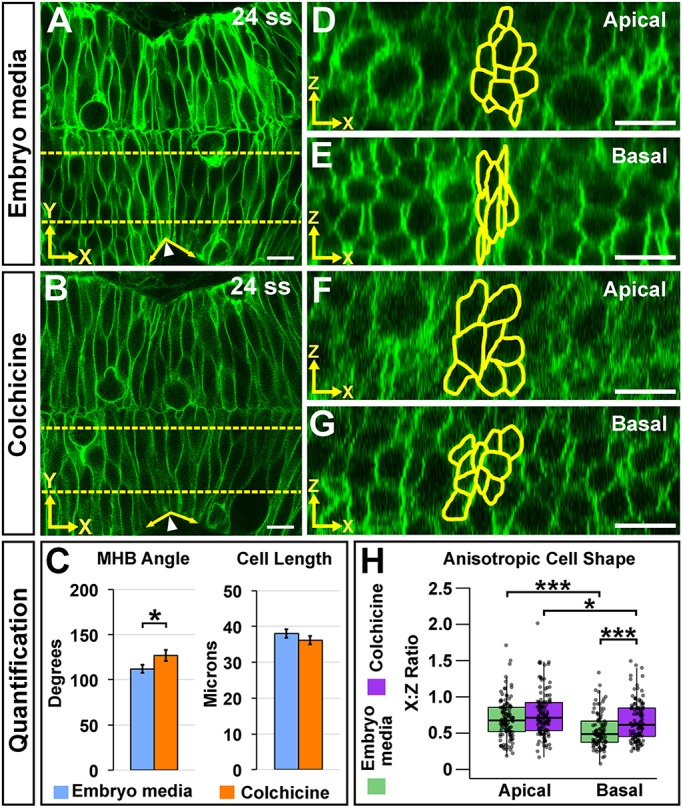
**Microtubule polymerization is required for basal anisotropic cell shape at the MHBC.** (A,B) Live confocal images of 24 ss memGFP-injected wild-type embryos treated at 18 ss with embryo media (A) or colchicine (B). (C) Quantification of MHB tissue angle and MHBC cell length. Data are represented as mean±s.e.m. of three independent experiments. (D,E) Apical (D) and basal (E) digital slices of embryo media-treated embryos. (F,G) Apical (F) and basal (G) digital slices of colchicine-treated embryos. MHBC cells are outlined in yellow. (H) Quantification of anisotropic cell shape using *x*:*z* ratio. Boxplots indicate the 25th and 75th percentiles and the median. Three independent experiments are represented. Control embryo media, *n*=7; colchicine, *n*=8. *P<0.05, ***P<0.005. Arrowheads indicate MHBC and arrows indicate MHB tissue angle. Scale bars: 10 μm.

Microtubules are well-established as important mediators of cell shape during neural tube formation (Burnside, 1971; Cearns et al., 2016; Jayachandran et al., 2016). As a next logical step in understanding the role of microtubules as a target for Wnt5b, we tested the hypothesis that disruption of microtubule polymerization would affect early MHB morphogenesis. We employed the widely used microtubule-destabilizing reagent colchicine (Dostál and Libusová, 2014; Ravelli et al., 2004), combined with live confocal imaging of memGFP-injected embryos. As predicted, colchicine treatment administered at 18 ss disrupted microtubule dynamics (Fig. S6A-C) and resulted in abnormal MHB tissue folding, but did not affect MHBC apical-basal cell length (Fig. 4A-C). Quantification of anisotropic cell shape revealed that preventing microtubule polymerization significantly increased the basal *x*:*z* ratio at the MHBC, with values closer to one (Fig. 4D-H). Apical anisotropic cell shape remained unchanged (Fig. 4D-H); however, we observed an increase in apical cell area (Fig. S6D) possibly due to an effect of colchicine on cell cycle progression and cell proliferation (Brues and Cohen, 1936; Ghawanmeh et al., 2018). In addition, colchicine did not affect cell shapes outside of the MHBC (Fig. S6E-I). Together, these data indicate that microtubule polymerization plays a region-specific role in the basal domain of MHBC cells to regulate anisotropic cell shape during early MHB tissue folding, which is independent from regulation of apical anisotropic cell shape.

### Microtubule filament stability is required for Wnt5b-mediated basal anisotropic cell shape

**Fig. 5.**
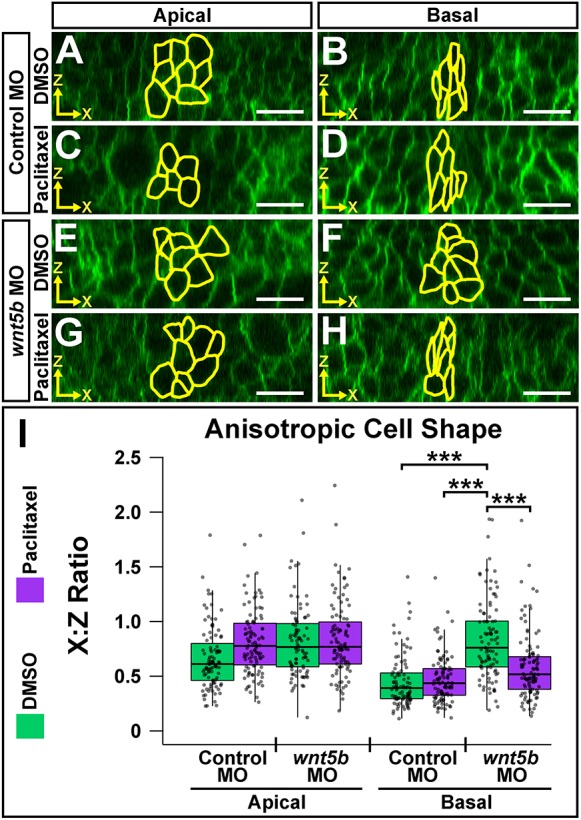
**Microtubule filament stability is required for Wnt5b-mediated basal anisotropic cell shape.** (A-H) Digital slices from *z*-series images of wild-type embryos co-injected with memGFP and control MO (A-D) or *wnt5b* MO (E-H). Embryos were treated at 18 ss with DMSO (A,B,E,F) or paclitaxel (C,D,G,H) and imaged at 24 ss. MHBC cells are outlined in yellow in apical (A,C,E,G) and basal (B,D,F,H) digital slices. (I) Quantification of anisotropic cell shape using *x*:*z* ratio. Apical measurements were compared post-hoc with the control MO DMSO apical condition. Basal measurements were compared post-hoc with the *wnt5b* MO DMSO basal condition. Boxplots indicate the 25th and 75th percentiles and the median. Four independent experiments are represented. Control DMSO, *n*=7; control paclitaxel, *n*=9; *wnt5b* MO DMSO, *n*=8; *wnt5b* MO paclitaxel, *n*=8. ***P<0.005. Scale bars: 10 μm.

We established that Wnt5b has a role in mediating apical and basal anisotropic cell shape, α-tubulin and β-tubulin levels, and microtubule dynamics within the MHB. We also demonstrated that microtubule polymerization is required specifically for basal anisotropic cell shape. These data led us to hypothesize that Wnt5b mediates MHBC anisotropic cell shape by regulating microtubule stability. To test this hypothesis, we performed a rescue experiment using paclitaxel, a microtubule-stabilizing reagent (Dostál and Libusová, 2014; Elie-Caille et al., 2007; Jayachandran et al., 2016), in combination with *wnt5b* knockdown. We predicted that stabilization of microtubules would rescue the *wnt5b* knockdown induced defects in anisotropic cell shape. Embryos were co-injected with memGFP and either control or *wnt5b* MO, then treated at 18 ss with DMSO or paclitaxel and imaged at 24 ss. Apical and basal digital slices were analyzed for anisotropic cell shape (Fig. 5). Control and *wnt5b* morphants treated with DMSO demonstrated apical and basal anisotropic cell shapes that were similar to those shown in Fig. 2 (Fig. 5A,B,E,F). However, when quantified, apical anisotropic cell shape of control morphants compared with *wnt5b* morphants was not significantly different (Fig. 5A,E,I). This may be due to the microtubule-polymerizing effect of DMSO treatment (Katsuda et al., 1987; Robinson and Engelborghs, 1982). Paclitaxel did not have a significant effect on control morphant anisotropic cell shape apically or basally (Fig. 5A-D,I). However, when we treated *wnt5b* morphants with paclitaxel, we found that basal anisotropic cell shape was rescued (Fig. 5F,H,I), and there was no effect on apical anisotropic cell shape (Fig. 5E,G,I). Together, these data indicate that basal anisotropic cell shape is specifically dependent on *wnt5b*-mediated microtubule stability.

### JNK is regulated by Wnt5b and mediates microtubule dynamics at the MHBC

**Fig. 6.**
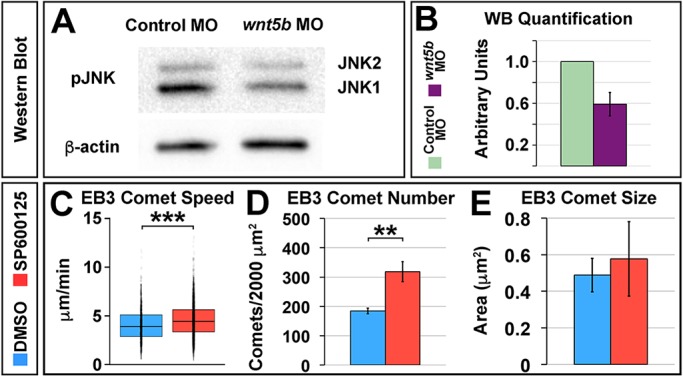
**JNK is regulated by Wnt5b and mediates microtubule dynamics at the MHBC.** (A) Representative western blot of phosphorylated JNK (pJNK) and β-actin levels from head tissue of control and *wnt5b* morphants. (B) Quantification of four independent western blot experiments, pJNK (JNK1 and JNK2) levels were normalized to β-actin. Data are represented as mean±s.d. (C-E) Quantification of EB3-GFP comet speed (C), EB3-GFP comet number (D) and EB3-GFP comet size (E). Boxplots indicate the 25th and 75th percentiles and the median. Three independent experiments are represented. In D,E, data are represented as mean±s.e.m. DMSO, *n*=6; SP600125, *n*=7. **P<0.01, ***P<0.005. See also Movies 5-8.

To examine what could mediate Wnt5b regulation of anisotropic MHBC cell shape, we investigated a known downstream effector of Wnt signaling that is also known to regulate microtubule dynamics, JNK (Schambony and Wedlich, 2007; Yang, 2003). JNK plays an important role in the brain during neurite growth and regeneration, notably through the phosphorylation of downstream MAPs (Feltrin et al., 2012). This known role for JNK, and our finding that Wnt5b regulates microtubules to mediate basal anisotropic cell shape, led us to hypothesize that JNK is regulated by Wnt5b during early MHB morphogenesis. We investigated the effect of *wnt5b* knockdown on JNK activation by examining levels of JNK phosphorylation (pJNK) (Fig. 6A,B). *wnt5b* knockdown decreased pJNK levels by approximately 40% (Fig. 6B), suggesting that JNK is a potential mediator of the Wnt5b signal.

Because we found that Wnt5b-mediated anisotropic cell shape is specific to the MHBC, we hypothesized that downstream effectors of the Wnt5b signal would also have subcellular specificity to elicit mechanistic differences in cell shape basally versus apically. It was previously demonstrated that inhibition of glycogen synthase kinase 3 beta (GSK3β), which normally targets β-catenin for degradation in the absence of Wnt signals, is sufficient to rescue the basal tissue-folding defect observed in *wnt5b* morphants (Gutzman et al., 2018). In addition, JNK cooperates with GSK3β to mediate microtubule stability (Ciani and Salinas, 2007) and studies have demonstrated a link between JNK and β-catenin localization (Lee et al., 2009; Liao et al., 2006). Therefore, we hypothesized that *wnt5b* could mediate the subcellular localization of β-catenin in MHBC cells. Immunohistochemistry revealed that *wnt5b* regulates basal, but not apical, accumulation of β-catenin and this basal accumulation of β-catenin is specific to the MHBC region (Fig. S7). These data suggest potential subcellular variations in Wnt5b signaling to mediate basal cell shape.

Next, we investigated whether or not JNK modulates microtubules at the MHBC by tracking EB3-GFP in either DMSO control or JNK inhibitor, SP600125 (He et al., 2016) treated embryos (Fig. 6C-E; Movies 5-8). Quantification of EB3-GFP comet speed, comet number and comet size were compared between treatments (Fig. 6C-E). We observed that SP600125 JNK inhibitor treatment resulted in a significant increase in EB3-GFP comet speed and comet number (Fig. 6C,D). However, we did not detect a significant difference in EB3-GFP comet size (Fig. 6E). These data are consistent with the microtubule effects observed following *wnt5b* knockdown (see Fig. 3I-K) and indicate that JNK regulates microtubule dynamics in the MHBC at 24 ss during basal tissue folding.

### JNK is required for basal anisotropic MHBC cell shape

**Fig. 7.**
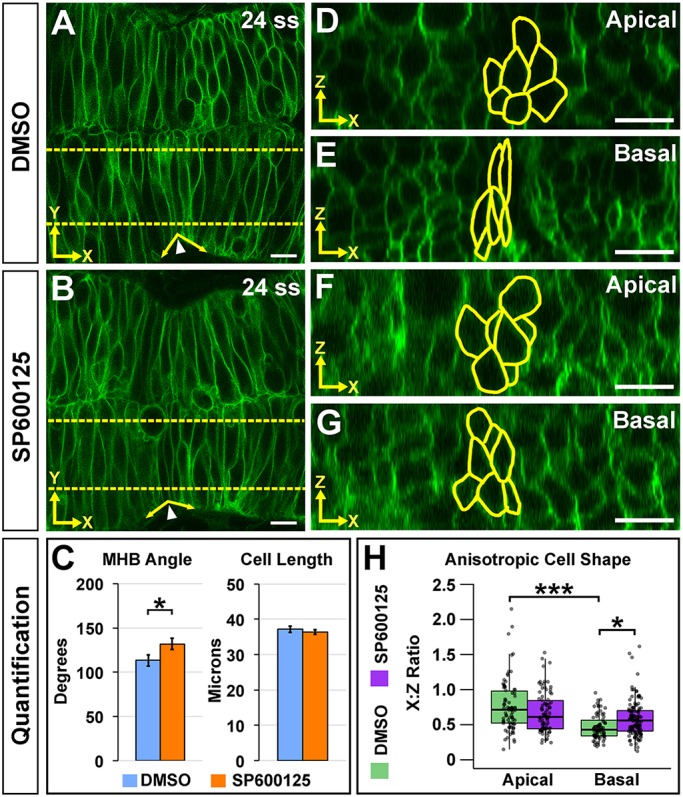
**JNK is required for basal anisotropic MHBC cell shape.** (A,B) Live confocal imaging of 24 ss embryos injected with memGFP and treated with DMSO (A) or SP600125 (B). (C) Quantification and comparison of MHB angle and length. Data are represented as mean±s.e.m. of three independent experiments. (D,E) Apical (D) and basal (E) digital slices of DMSO-treated embryos at 24 ss. (F,G) Apical (F) and basal (G) digital slices of SP600125-treated embryos at 24 ss. MHBC cells are outlined in yellow in apical (D,F) and basal (E,G) digital slices. (H) Quantification of anisotropic cell shape using *x*:*z* ratio in DMSO- and SP600125-treated embryos. Boxplots indicate the 25th and 75th percentiles and the median. Three independent experiments are represented. DMSO, *n*=6; SP600125, *n*=6. *P<0.05, ***P<0.005. Arrowheads indicate MHBC and arrows indicate MHB tissue angle. Scale bars: 10 μm.

We demonstrated that Wnt5b regulates microtubules to establish basal anisotropic cell shape. We have also shown that Wnt5b regulates JNK, and that JNK modulates microtubule dynamics at the MHBC. Therefore, we hypothesized that JNK would also be required for MHB tissue and cell shape. We treated memGFP-injected embryos with DMSO control or the JNK inhibitor SP600125 at 18 ss and analyzed MHB tissue and cell shape at 24 ss (Fig. 7). SP600125 treatment perturbed the MHB angle, indicating that JNK has a role in MHB basal tissue folding (Fig. 7A-C), but not via mediation of apical-basal cell length (Fig. 7C). Next, we characterized anisotropic cell shape using the *x*:*z* ratio from apical slices and did not detect a significant difference with JNK inhibition (Fig. 7D,F,H). However, when we quantified anisotropic cell shape in basal slices, JNK inhibition resulted in a significant increase in the *x*:*z* ratio (Fig. 7E,G-H). Differences in anisotropic cell shape were not observed in cells outside the MHBC with JNK inhibition (Fig. S8). Together, these results indicate that JNK is required for MHB basal tissue folding through specific modulation of MHBC basal anisotropic cell shape.

### Microtubule filament stability is required for JNK-mediated basal anisotropic cell shape

**Fig. 8.**
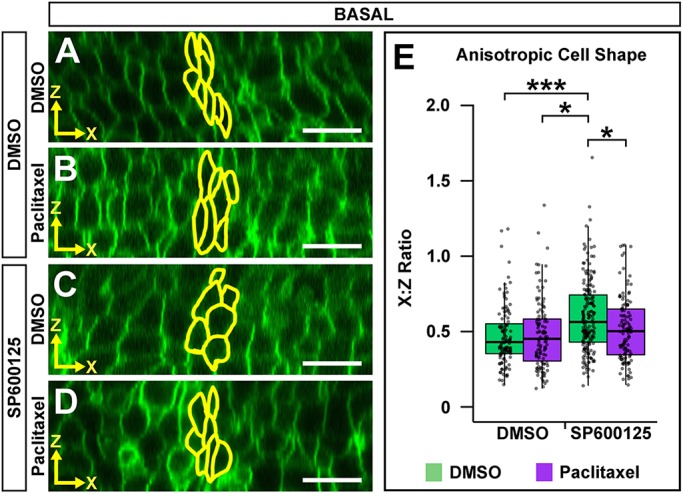
**Microtubule filament stability is required for JNK-mediated basal anisotropic cell shape.** (A-D) Digital slices from *z*-series images of memGFP-injected wild-type embryos treated at 16 ss with DMSO (A,B) or SP600125 (C,D). Embryos were then treated at 18 ss with DMSO (A,C) or paclitaxel (B,D) and imaged at 24 ss. MHBC cells are outlined in yellow. (E) Quantification of anisotropic cell shape. All basal *x*:*z* ratios were compared post-hoc with the SP600125/DMSO treatment. Boxplots indicate the 25th and 75th percentiles and the median. Four independent experiments are represented. DMSO/DMSO *n*=6; DMSO/paclitaxel *n*=6; SP600125/DMSO *n*=10; SP600125/paclitaxel *n*=7. *P<0.05, ***P<0.005. Scale bars: 10 μm.

We have shown that JNK is required for mediating microtubule dynamics and basal anisotropic cell shape at the MHBC to control MHB basal tissue folding. These data led us to hypothesize that JNK mediates basal MHBC anisotropic cell shape by regulating microtubule stability as we have shown with Wnt5b. To test this hypothesis, we performed a rescue experiment using paclitaxel in combination with the JNK inhibitor SP600125. Wild-type embryos were injected with memGFP, treated at 16 ss with either DMSO or SP600125, and then treated at 18 ss with DMSO or paclitaxel. At 24 ss, embryos were imaged and basal anisotropic cell shape was analyzed (Fig. 8). Control and SP600125-treated embryos, which were additionally treated with DMSO at 18 ss, demonstrated basal anisotropic cell shapes similar to the treatment with JNK inhibitor presented in Fig. 7 (Fig. 7E,G and Fig. 8A,C). However, when we treated SP600125-treated embryos with paclitaxel, we found that basal anisotropic cell shape was rescued (Fig. 8C,D,E). Together, these data indicate that basal MHBC anisotropic cell shape is dependent on JNK-mediated microtubule stability.

## DISCUSSION

**Fig. 9.**
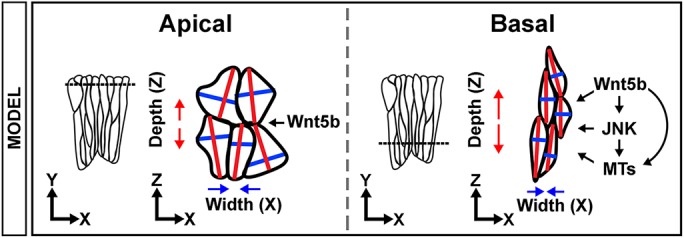
**Proposed mechanism for differential regulation of apical and basal anisotropic cell shape in MHBC cells.** Apically, MHBC cells are anisotropic, where cells are narrower in the anterior-posterior (*x*) direction (blue arrows) and deeper in the dorsal-ventral (*z*) direction (red arrows). This cell shape is dependent on Wnt5b. Basally, Wnt5b, JNK and microtubules (MTs) all regulate anisotropic cell shape. Wnt5b and JNK regulation of basal MHBC cell shape is microtubule dependent. Together, our model suggests that Wnt5b is likely to mediate JNK activation, and in turn affect microtubule stability and dynamics, to specifically regulate basal anisotropic cell shape during early MHB morphogenesis.

From these studies, we propose a mechanistic model for the differential regulation of apical versus basal MHBC anisotropic cell shape at 24 ss to form the basal MHB tissue fold (Fig. 9). Apically, our data indicate that Wnt5b functions to maintain anisotropic cell shape in the width (*x*-axis) and depth (*z*-axis) dimensions. However, we found a different role for Wnt5b, and other downstream factors, basally (Fig. 9). On the basal side of the MHBC cells, Wnt5b, JNK and microtubules are each required to decrease the *x*:*z* ratio and to enhance changes in anisotropic cell shape. This enhancement is in turn required to fold the MHB epithelial tissue along the basal side in the anterior-posterior (*x*-axis) direction. These new data are consistent with our previous finding that narrowing of MHBC cells in the width (*x*-axis) direction is required for acute anterior-posterior fold formation. Here, we propose that deepening of cells in the depth (*z*-axis) dimension may be required to restrict the overall tissue shape to generate the sharp MHB fold in two dimensions. In contrast to the apical side of the cell, Wnt5b regulation of basal MHBC cell shape is microtubule dependent. In addition, JNK modulates microtubule dynamics in a similar manner at the MHBC and JNK regulation of basal MHBC cell shape is also microtubule dependent. Collectively, our data suggest a model in which Wnt5b mediates JNK activity, which affects microtubule dynamics to regulate specifically basal anisotropic cell shape. We have shown that a single morphogen, Wnt5b, modulates both apical and basal anisotropic cell shape within the same cellular context, but through independent mechanisms whereby Wnt5b-mediated regulation of basal, but not apical, anisotropic cell shape is microtubule dependent. Although we know that anisotropic cell shape, basally, at the MHBC is determined by Wnt5b-mediated microtubule stability, the exact mechanism for how Wnt5b specifically elicits this anisotropic cell shape is not yet known.

Microtubules have long been associated with regulation of cell and tissue shape changes, such as neuroepithelial cell elongation (Burnside, 1971; Cearns et al., 2016; Jayachandran et al., 2016; Messier, 1969). Microtubules are also important for apical constriction in fly eye disc epithelium and salivary gland development (Booth et al., 2014; Fernandes et al., 2014), and in bottle cells during *Xenopus* gastrulation (Choi and Sokol, 2009; Lee and Harland, 2007). However, in bottle cells, microtubules were not found to regulate cell length, as they do in cells undergoing neurulation (Lee and Harland, 2007). Similarly, we found that microtubules did not regulate cell length in MHBC cells. This demonstrates that the role of microtubules in mediating different aspects of cell shape is cell context specific. When we perturbed microtubule polymerization with colchicine, or when we stabilized microtubules using paclitaxel in *wnt5b* knockdown embryos, basal, but not apical, MHBC cell shape was affected. Consistent with this, during these early stages of MHB morphogenesis, MHBC apical cell shape did not change. However, basal cell shape was more polarized and changed over time, suggesting that earlier stages of MHB folding differentially rely on microtubule-mediated basal cell shape changes. During late MHB formation, at 24 hours post-fertilization, microtubules are also likely to play a role in basal constriction (Graeden, 2011).

One possible mechanism for how Wnt5b could modulate apical versus basal cell shape is by regulating apical- and basal-specific proteins to polarize the cells. Shroom3, N-cadherin and Vangl2 are all apically localized and are required for apical constriction (Morita et al., 2010; Ossipova et al., 2015; Plageman et al., 2011), whereas basally localized integrins, focal adhesion kinase (FAK) and the basement membrane are required for basal constriction (Bogdanović et al., 2012; Gutzman et al., 2018, 2008). Although there have been numerous investigations of apically localized proteins in the context of planar cell polarity (PCP) and regulation of apical cell shape, there are few studies describing the mechanisms by which basally localized proteins mediate basal cell shape. Here, we demonstrate that β-catenin, a Wnt signaling variant, is enriched basally and specifically at the MHBC, in a *wnt5b*-mediated manner. Based on these data, we propose that Wnt5b regulation of microtubule stability is likely to be required for its polarizing activity within the basal domain of the MHBC cells, which is in turn required for the MHB basal tissue fold (Gutzman et al., 2018).

During MHB basal tissue folding, we also found that Wnt5b modulates JNK activity, a known downstream target of non-canonical Wnt signaling (Komiya and Habas, 2008; Yamanaka et al., 2002; Yang, 2003). We also observed that JNK is necessary for proper basal, but not apical, cell shape and for microtubule dynamics. In addition, inhibition of GSK3β, a kinase associated with canonical Wnt signaling, is also sufficient for Wnt5b-mediated tissue folding (Gutzman et al., 2018). This suggests a role for both canonical and non-canonical Wnt signaling in mediating MHB morphogenesis. In addition, both JNK signaling and GSK3β inhibition have been observed within the same cellular context to promote microtubule stability (Ciani and Salinas, 2007). Therefore, we postulate that the observed higher number and greater speed of EB3-GFP comets shown here with knockdown of *wnt5b* and with JNK inhibition, may be due to reduced microtubule stability within the MHBC region and be indicative of a compensatory response to the reduction of total microtubules. Together, these observations lead us to hypothesize that Wnt5b regulation of JNK, and potentially GSK3β, mediate basal anisotropic cell shape through modulation of MAPs, such as microtubule-associated protein 1B (MAP1B) (Chang et al., 2003; Ciani and Salinas, 2007; Feltrin et al., 2012; Gutzman et al., 2018; Jayachandran et al., 2016).

JNK signaling is also a modulator of actomyosin activity. Studies have demonstrated a crucial role for the actomyosin network in modulating apical and basal constriction (Gutzman et al., 2015; Martin et al., 2009; Nishimura et al., 2012). One obvious possibility is that Wnt5b affects basal cell shape via regulation of the actomyosin network. Interestingly, our studies did not reveal a direct link between Wnt5b signaling and the actomyosin network (Fig. S5). Upon re-analysis of our previously published data using the orthogonal slice method presented here, we found that non-muscle myosin IIB is required for basal anisotropic cell shape (M.R.V. and J.H.G., unpublished results) (Gutzman et al., 2015). These data confirm a role for the actomyosin network in mediating these cell shape changes, which appear to be Wnt5b independent. However, we cannot exclude the possibility that Wnt5b-mediated microtubule stability is required to poise the cells to respond to mechanotransduction signals, which could be mediated through the extracellular matrix (Gutzman et al., 2008). Additional examination of potential crosstalk with other signaling pathways will be necessary to identify the specific functions of Wnt5b-mediated microtubule stability, which are likely to be multifactorial.

As we know that Wnt signaling and microtubules are important for polarized PCP protein localization (Matis et al., 2014; Sepich et al., 2011) and planar polarization of actomyosin networks to regulate apical constriction (Nishimura et al., 2012), it is also possible that Wnt5b mediates trafficking of cargo basally within the *xz* plane of MHBC cells to control basal MHB folding. Alternatively, microtubules may be required for trafficking of other protein complexes on the basal side of the cells to confer basal tissue folding. It is also possible that re-distribution of basal cell membrane via endocytic pathways is important for basal tissue folding; however, it is not likely to be an early mechanism for mediating basal cell shape as we demonstrate that basal cell area does not change at these early time points. The exact mechanisms for how Wnt5b differentially modulates apical versus basal cell shape remain unknown and will be the focus of future studies.

To form the basal epithelial tissue fold that generates the MHB during development we know that cells must shorten, become narrower, expand apically and constrict basally (Gutzman et al., 2008, 2015). Here, we have revealed that basal tissue folding also requires a change in basal anisotropic cell shape at the deepest point of the fold. Although we have begun to uncover the underlying mechanisms for controlling this basal fold, major questions remain. How are apical versus basal cell shape changes independently coordinated? What is the mechanism for Wnt5b modulation of microtubules? How does microtubule stability lead to changes in basal cell shape? Understanding both the cellular and molecular mechanisms that coordinate differential cell and tissue shaping are crucial for the understanding of developmental disease and for the future of tissue engineering and regenerative medicine.

## MATERIALS AND METHODS

### Zebrafish husbandry, maintenance and strains

Zebrafish (*Danio rerio*) embryos were used for these studies and include wild type (AB) and *wnt5b* mutants, *pipetail^(ti265)^* (Hammerschmidt et al., 1996). Zebrafish husbandry, maintenance and embryo care were performed according to Westerfield (2007). For all experiments, embryo stage was determined according to somite number following standard guidelines (Kimmel et al., 1995). Somite number was utilized to account for any possible developmental delays. This study was conducted under the approval and supervision of the University of Wisconsin-Milwaukee Institutional Animal Care and Use Committee.

### mRNA and morpholino injections

Microinjections were performed at the one-cell stage using the following reagents and concentrations. For mRNA injections: CAAX-eGFP (memGFP), 150 pg/embryo; membrane Cherry (memCherry), 50 pg/embryo; EMTB-GFP, 100 pg/embryo; EB3-GFP, 100 pg/embryo. All mRNA was synthesized using SP6 mMessage mMachine Transcription Kit (AM1340, Ambion). EMTB-GFP and EB3-GFP constructs were kindly provided by Dr Caren Norden (Max Planck Institute of Molecular Cell Biology and Genetics, Dresden, Germany) (Norden et al., 2009). For antisense MO-mediated knockdown experiments: standard control MO (5′-CCTCTTACCTCAGTTACAATTTATA-3′), 3 ng/embryo; zebrafish *p53* MO (5′-GCGCCATTGCTTTGCAAGAATTG-3′), 3 ng/embryo (Robu et al., 2007); splice site-blocking *wnt5b* MO (5′-TGTTTATTTCCTCACCATTCTCCG-3′) 3 ng/embryo (De Rienzo et al., 2012; Gutzman et al., 2018; Robu et al., 2007; Young et al., 2014). For all MO experiments, *p53* MO was co-injected. All MO oligonucleotides were obtained from (Gene Tools, LLC). MO-injected embryos are referred to as morphants.

### Live confocal imaging and cell shape analysis

Live confocal imaging was carried out as previously described using a Nikon C2 laser scanning confocal and a 40× water-immersion objective lens (NA 1.15) at room temperature (Gutzman et al., 2015; Sahu et al., 2017). Embryos were oriented on a microscope slide in 1% agarose wells with the anterior region of the embryo facing the left and posterior region facing the right. The dorsal side of the embryo, at the MHB, was positioned closest to the cover slip such that the midline of the embryo was parallel to the *x*-axis and the MHBC was centered. This was done to ensure consistency in tissue and cellular orientation when imaged and quantified for each embryo. Images were acquired in a *z*-series spanning 15-25 μm in depth for each embryo. Stacks were collected starting 15 μm below the dorsal surface at the MHB. Each figure contains representative images of a single slice from a *z*-series (*xy* plane) or a view of the projected *xz* plane utilizing Nikon Imaging System (NIS) Elements Software to acquire a digital orthogonal cross-section of the *z*-series (Digital slice). All confocal images were processed and analyzed using NIS Elements, Fiji (ImageJ) or Photoshop (Adobe). Cell length (*y*-axis) measurements and MHB angle analyses were performed as previously described (Gutzman et al., 2015; Sahu et al., 2017).

To ensure that cells within the image were consistently aligned for proper quantification of anterior-posterior (*x*-axis) cell width and dorsal-ventral (*z*-axis) cell depth, the following cell alignment protocol was followed. First, *z*-stacks of data were rotated in the *xy* plane to orient the apical-basal length of MHBC cells parallel to the *y*-axis. Next, *xz* digital sections were taken from the newly oriented *z*-stack both apically and basally perpendicular to the length of the cell (*y*-axis). This allowed for generation of *xz* plane digital sections that aligned the same MHBC cells in both the apical and basal *xz* images. In order to measure cell length, we identified one MHBC cell at the deepest point of constriction within the confocal *z*-series dataset that clearly spanned the entire distance from the apical surface of the tissue at the midline to the basal surface of the tissue and took a *y*-axis measurement (Gutzman et al., 2015). MHBC cell length was then divided into six equal sections along the apical-basal cell axis. Using these sections as guides, two digital slices in the *xz* plane were generated from the NIS Elements Software Slices View Module. On each side of the neural tube, one slice was generated at the section point immediately below the apical cell surface and one slice was generated immediately above the basal surface. On average, these digital slices were about 5-7 μm into the cell from the apical or basal cell surface (see Fig. 1D-L; Fig. S1). Apical and basal digital slices were then used for the quantification of apical and basal *x*:*z* cell shape. The dashed yellow line shown in each figure indicates apical and basal section positions, where digital slices were generated.

Three measurements were acquired for cell shape analysis from each digital slice: cell area, *z*-dimension (depth) and *x*-dimension (width). Cell area was quantified using the free-hand selection tool in Fiji to outline individual cells in the *xz* plane. Each cell outline was then defined as a region of interest (ROI). In the ROI manager, cell area was calculated from the ROIs in μm. The *z*-dimension of the MHBC cells was measured using the line and measure tool along the deepest point of the cell, within 45° of the *z*-axis. The *x*-dimension was measured at the widest point of the cell, perpendicular to the *z*-dimension measurement (see Fig. 1C; Fig. S1D″,E″). To quantify anisotropic cell shape, the *x*-dimension measurement was divided by the *z*-dimension measurement resulting in an *x*:*z* ratio. Data are presented as *x*:*z* ratios. Ratios with values that are greater than one, or less than one, indicate anisotropic, or polarized, cell shape.

### Microtubule imaging and analysis

We examined microtubule filaments with live confocal imaging of EMTB-GFP, and plus-end microtubule dynamics with live confocal imaging of EB3-GFP. We quantified filament intensity, comet number, comet size and comet speed. Analyses were conducted on wild-type embryos that had been co-injected with memCherry mRNA, EB3-GFP, or EMTB-GFP mRNA either alone, or combined with control MO or *wnt5b* MO.

To quantify microtubule filaments, EMTB-GFP-injected embryos were live imaged at 24 ss. We used a Nikon C2 laser scanning confocal microscope and collected a 15-25 μm *z*-series of images. The stack was cropped to 10 μm and was analyzed using the average intensity projection of a *z*-series in Fiji. To quantify MHBC-specific EMTB-GFP intensity, 40 μm×50 μm ROIs were drawn using the rectangle selection tool and analyzed using the measure tool. ROIs were drawn at the MHBC, immediately adjacent to the MHBC in the midbrain region, and immediately adjacent to the MHBC in the hindbrain region (see Fig. S4A-C). An intensity measurement of each region was obtained using Fiji. Each side of the neuroepithelium was analyzed for each embryo. To compare MHBC average intensity across embryos and experiments, we divided the MHBC average intensity by the average of the intensity at the midbrain and hindbrain within each individual embryo, then we compared multiple embryos under different conditions (with or without *wnt5b* knockdown). This ratio is termed the normalized MHBC EMTB-GFP intensity (see Fig. S4C).

To quantify the plus-ends of microtubules, EB3-GFP-injected embryos were imaged at 21-24 ss. We used a Nikon C2 laser scanning confocal microscope and imaged in a single plane, 15-20 μm into the tissue from the dorsal side of the embryo. Time-lapse data were collected for 10 min at a scanning speed of 1 frame per 4 s. Data were then cropped to 200 s and analyzed for EB3-GFP comet speed, comet number and comet size using Fiji software. In Fiji, each image series was cropped to equal size (50 μm×40 μm) in the region at the MHBC on one side of the neural tube at a time. The ROI used for EB3-GFP analysis did not include the apical region of the cells to avoid quantification of microtubules within dividing cells and to avoid the mitotic organizing center population of microtubules at the apical midline. Next, each image series was converted into 8-bit gray scale. The cropped data were segmented for EB3-GFP comets using the Threshold plugin and Otsu method, followed by the Analyze Particle plugin. EB3-GFP comets were then analyzed using the Fiji TrackMate plugin (Szikora et al., 2017). Particle detection was performed using the Laplacian of Gaussian detector method with an estimated blob diameter of 1 μm and a threshold of 0.5 μm with sub-pixel localization. Auto initial thresholding of spot quality was used to filter spots. Tracks were detected using the Linear motion LAP Tracker (see Movies 1-8).

### Immunohistochemistry

For β-tubulin immunostaining, embryos were fixed at 24 ss in 4% paraformaldehyde with 80 mM KPIPES, 5 mM EGTA, 1 mM MgCl_2_ and 0.2% Triton X-100, pH 6.4, for 5 min at 28°C, followed by 3 h at room temperature and then washed with Tris-buffered saline and 0.1% NP-40 (TBS-NP40) (155 mM NaCl, 10 mM Tris-HCl and 0.1% NP-40, pH 7.6), three times for 10 min each at room temperature. Embryos were then de-yolked and blocked (5% normal goat serum, 2% bovine serum albumin, 1% Triton X-100 in TBS-NP40) for 1 h at room temperature. After blocking, embryos were incubated in β-tubulin antibody (1:200, E7-5, Developmental Studies Hybridoma Bank) in blocking solution for 72 h at 4°C and then washed three times in TBS-NP40 for 10 min each at room temperature. After the embryos were washed, they were incubated in Alexa Fluor 555-conjugated secondary antibody (1:500, A21422, Invitrogen) in blocking solution overnight at 4°C. After three washes in TBS-NP40 for ten minutes each, embryos were flat-mounted in glycerol and imaged using a Nikon C2 confocal with a 60× oil immersion objective lens. Quantification was carried out as follows: β-tubulin average intensity was quantified in a 10 µm^2^ box for apical, middle and basal regions of the MHBC within each embryo (Fig. S4D-F). The average intensity at the basal and apical MHBC region was divided by the average intensity at the middle MHBC region in the same embryo. This normalization was used for comparison of basal and apical MHBC intensity across embryos and for comparisons between control MO- and *wnt5b* MO-injected embryos.

### Western blot analysis

For western blot analysis, 22-24 ss embryos were dechorionated in embryo media and head tissue was dissected using fine-tip forceps. Tissue was collected in 1.5 ml Eppendorf tubes on ice containing buffer comprising 25 mM Tris (pH 8.0), 2 mM EDTA (pH 8.0), 10% glycerol, 1% Triton-X-100, phosphatase inhibitor (88667, Pierce) and protease inhibitor (04693124001, Roche). Total protein was isolated, and concentration measured by Bradford assay. Proteins (25 µg) were separated on 4-20% gradient SDS-PAGE and subsequent western blot analysis was conducted with the following conditions. Blots were blocked for 2 h at room temperature in 5% milk in 1× TBST (Tris-buffered saline with 0.1% Tween-20), then washed 3×30 min in 1× TBST and incubated overnight at 4°C in primary antibody. pJNK and β-tubulin primary antibodies were diluted in 5% milk in 1× TBST; all other primary antibodies were diluted in 0.25% gelatin in 1× TBST. The next day, blots were washed 3×30 min in 1× TBST and incubated for 1 h in secondary antibody in 5% milk in 1× TBST. Following secondary antibody incubation, blots were washed and imaged using ECL western blotting substrate (1705060, Bio-Rad). Primary antibodies used were: α-tubulin (1:2000, T6199, Sigma-Aldrich), β-tubulin (1:2000, E7-5, Developmental Studies Hybridoma Bank), phosphorylated-JNK (pJNK) (1:1000, 4668, Cell Signaling Technology), β-actin (1:2000, A5441, Sigma-Aldrich). Secondary antibodies used were: anti-mouse HRP (1:2000, 7076, Cell Signaling Technology) and anti-rabbit HRP (1:2000, 7074, Cell Signaling Technology). Blots were imaged using a Syngene G:BOX Chemi XRQ imaging system.

### Pharmacological manipulations

For all pharmacological manipulations, embryos were dechorionated at 15-17 ss and placed in 1% agarose-coated Petri dishes containing embryo media. Normal media was then replaced with embryo media containing pharmacological reagents and embryos were bathed in the treatment from either 16-24 ss or 18-24 ss, as indicated per experiment, then live imaged at 24 ss. Concentrations of reagents are as follows: JNK inhibitor SP600125 (420119, Calbiochem) was solubilized in DMSO and used at a final concentration of 5 μM; colchicine (C9754, Sigma-Aldrich) was solubilized in water and used at a final concentration of 100 μM; paclitaxel (T7191, Sigma-Aldrich) was solubilized in DMSO and used at a final concentration of 100 μM. All DMSO control treatments utilized the same volume percentage of DMSO as the compared pharmacological reagent treatment group.

### Statistical analysis

R-3.4.2 was used for all statistical analyses. Statistical comparisons between two groups were conducted using the Welch's *t*-test. For analyses of experiments with more than two treatment groups, one-way ANOVA was performed with experimental batch effect factored in. ANOVA analyses that yielded a *P*-value less than 0.05 were subjected to Tukey's Honest Significant Difference (HSD) post-hoc tests to determine significant differences between each of the treatment groups. For each figure, asterisks indicate statistical significance as follows: *P<0.05, **P<0.01, ***P<0.005. In each figure legend, *n* represents the number of embryos and the number of independent experiments conducted is presented. All box and whisker plots and scatter plot overlays were generated using the ggplot2 package in R. All other bar graphs were generated using Excel (Microsoft).

## Supplementary Material

Supplementary information
