# Supplementary material for: Basal epithelial tissue folding is mediated by differential regulation of microtubules
Source: Development. 2018 Nov 19;145(22):dev167031. doi: 10.1242/dev.167031 (PMC6262788; doi:10.1242/dev.167031)
Supplement: Supplementary information [file develop-145-167031-s1.pdf]

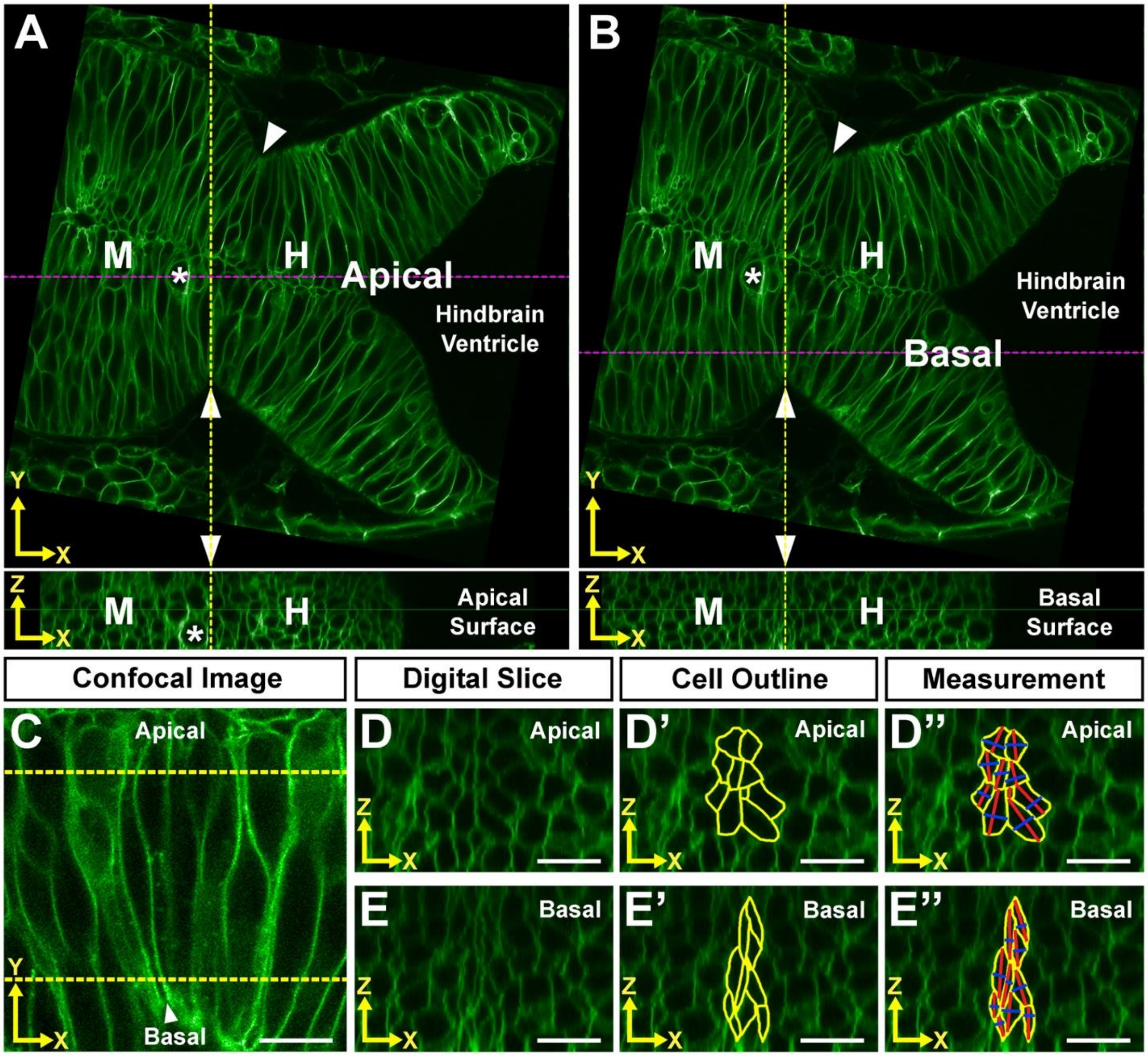

Fig. S1. Z-series processing of live confocal images to project data in the X-Z plane and to quantify anisotropic cell shape. (A,B) NIS Elements Software Digital Slices View Module of Apical (A) and Basal (B) slices in a Wild-Type (WT) embryo. M, midbrain. H, hindbrain. Arrowhead indicates MHBC. Dotted yellow line indicates position of MHBC in X-Y and X-Z plane views. Dotted purple line indicates position where digital orthogonal slices were acquired from the X-Y image. * indicates proliferative cell. (C) Live confocal imaging of 24 ss wild-type embryo injected with memGFP mRNA. Yellow dotted lines indicate position of apical and basal digital slices. (D,D'') Digital slice of Z-series at the apical end of the cell. (E') Apical MHBC cells outlined in yellow. (D'') Representative measurements of cell width (X) (red lines), and cell depth (Z) (blue lines). (E,E'') Digital slice of Z-series at the basal end of the cell. (F') Basal MHBC cells outlined in yellow. (E'') Representative measurements of cell width (X) and cell depth (Z). Arrowhead indicates MHBC. Anterior is to the left in all images. Scale bars: 10 μm.

Development • Supplementary information

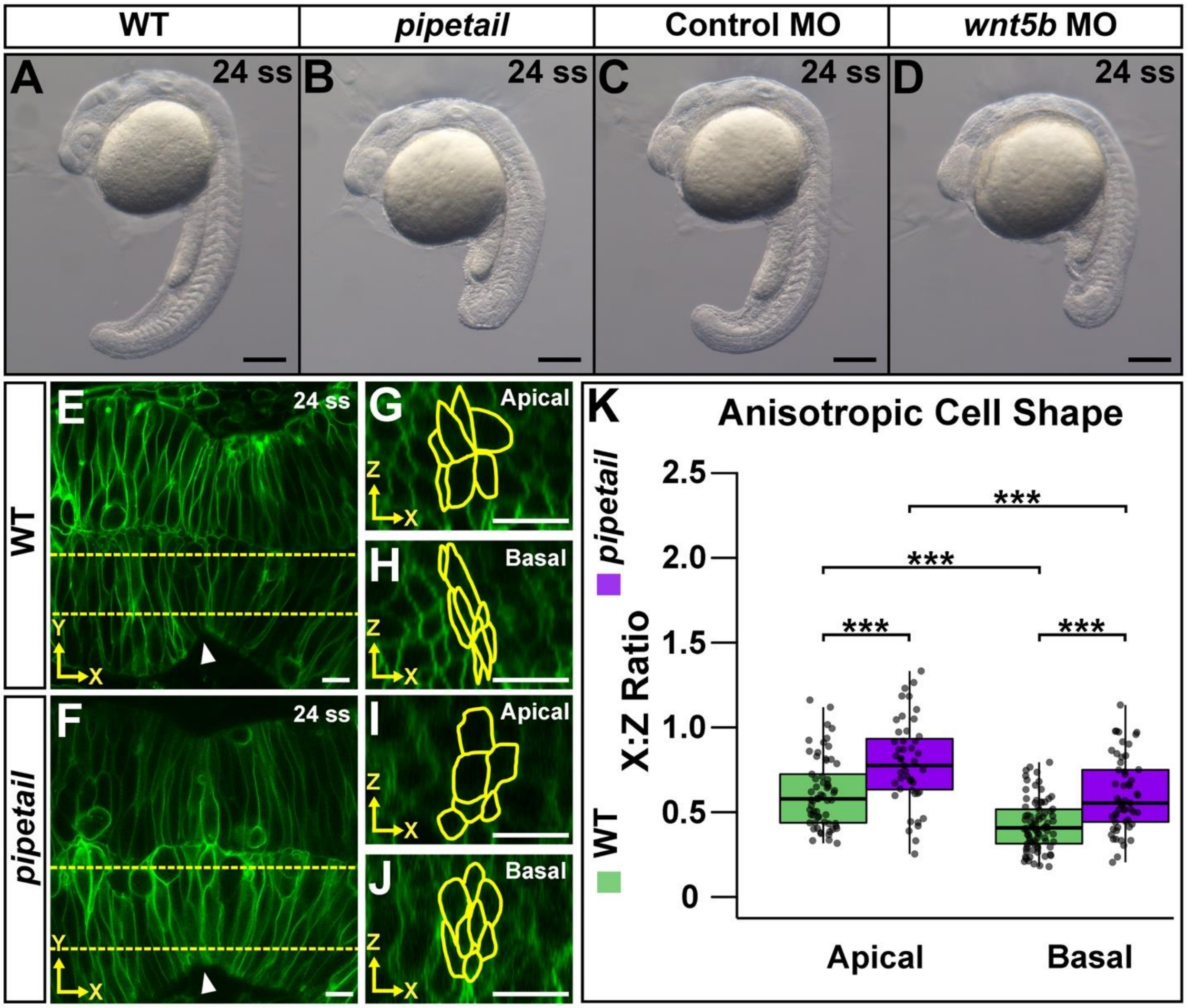

Fig. S2. *wnt5b* morpholino knockdown phenocopies *wnt5b pipetail$^{(ti265)}$* mutants, including defects in anisotropic cell shape at the MHBC. (A-D) Lateral brightfield images of 24 ss Wild-Type (WT) (A), *pipetail* (B), Control morphant (C), or *wnt5b* morphant (D) embryos. Scale bars: 200 µm. (E-F) Live confocal imaging of 24 ss WT (E) or *pipetail* (F) embryos injected with memGFP. (G-H) Apical (G) and basal (H) digital slices of WT embryos at 24 ss. (I,J) Apical (I) and basal (J) digital slices of *pipetail* mutant embryos at 24 ss. (K) Quantification of X:Z ratio. Box plots indicate the 25th and 75th percentiles and the median. Three independent experiments are represented. WT, *n=6*; *pipetail, n=4*. *** indicates *P*<0.005. Arrowhead indicates MHBC. Scale bars: 10 µm.

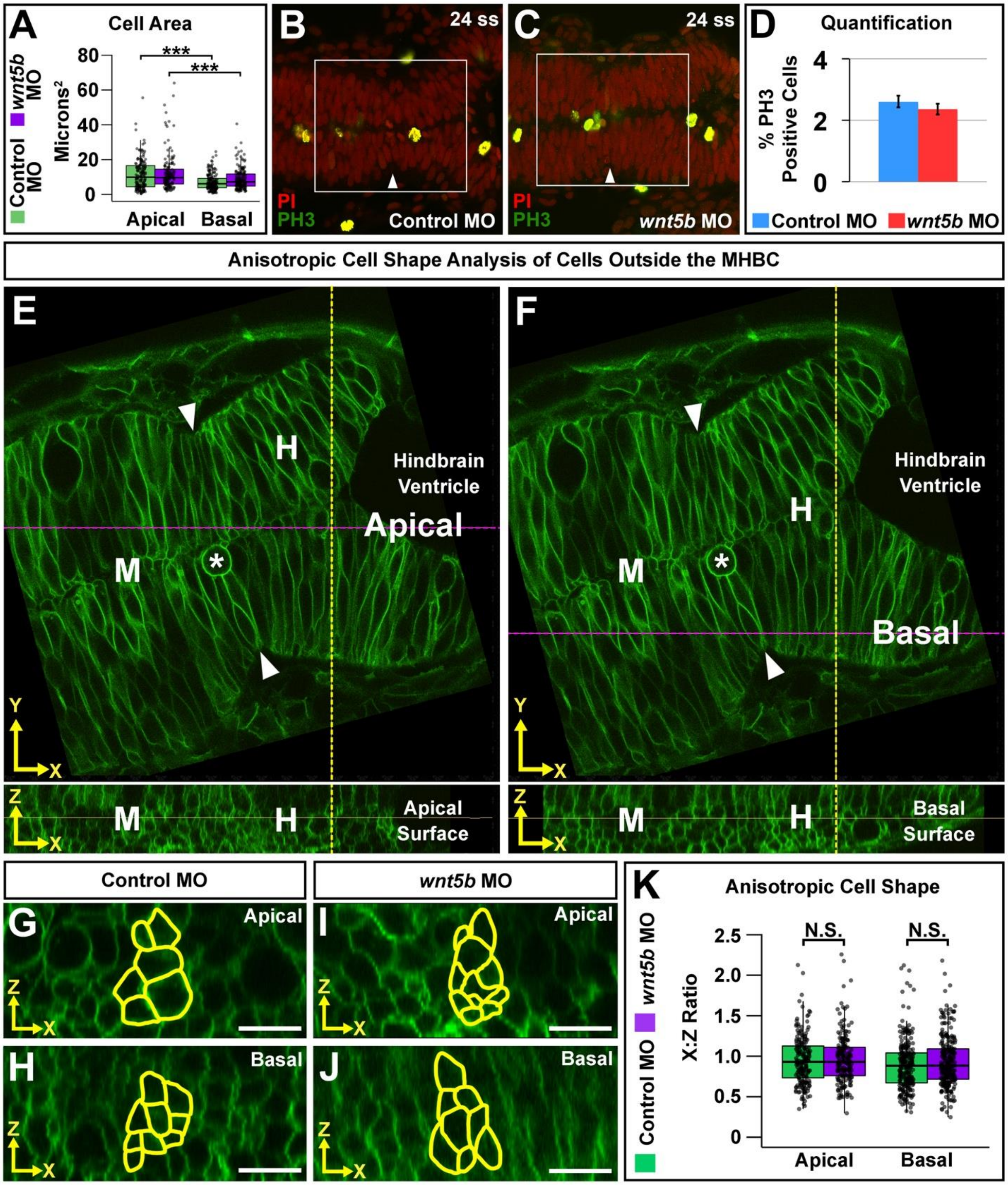

Fig. S3. *wnt5b* knockdown does not affect apical or basal MHBC cell area, MHB cell proliferation, or anisotropic cell shape outside of the MHBC during early morphogenesis. (A) Quantification of apical and basal cell area of 24 ss wild-type embryos co-injected with memGFP mRNA and Control or *wnt5b* MO. Boxplots indicate the 25th and 75th percentiles and the median. Eight independent experiments are represented. *** indicates P<0.005. Control MO, n=10; *wnt5b* MO, n=10. (B-D) Confocal images were acquired of Control MO (B) and

Development • Supplementary information

wnt5b MO (C) injected embryos. Embryos were fixed at 24 ss in 4% paraformaldehyde in phospho-buffered saline with 0.1% Tween-20 (PBT) for 2 hours and immunostained for phospho-histone 3 (1:800, Millipore, #06-570) and counter-stained with propidium iodide. (D) Quantification of PH3 positive cells as a percentage of total cells in the region indicated by the white box. Data represented as mean ± SEM from three independent experiments. Control MO, *n*=3; *wnt5b* MO, *n*=3. (E,F) Z-series processing of live confocal images to quantify anisotropic cell shape in cells outside of the MHBC, 40 microns posterior. NIS Elements Software Digital Slices View Module of Apical (E) and Basal (F) slices in a 24 ss WT embryo. M, midbrain. H, hindbrain. Arrowhead indicates MHBC. Dotted yellow line indicates position of cells that are 40 microns posterior to the MHBC in X-Y and X-Z plane views. Dotted purple line indicates position where digital orthogonal slices were acquired from the X-Y image. * indicates proliferative cell. (G-K) Anisotropic cell shape outside of the MHBC was not different between Control and *wnt5b* morphants. Apical (G,I) and basal (H,J) digital slices of WT 24 ss embryos co-injected with memGFP and either Control MO (G,H) or *wnt5b* MO (I,J). Cells that are 40 microns posterior to the MHBC are outlined in yellow. (K) Quantification of X:Z ratio. Box plots indicate the 25th and 75th percentiles and the median. Eight independent experiments are represented. Control MO, *n=10*; *wnt5b* MO*, n=10*. N.S. (*not significant*) indicates *P*>0.05. Scale bars: 10 µm.

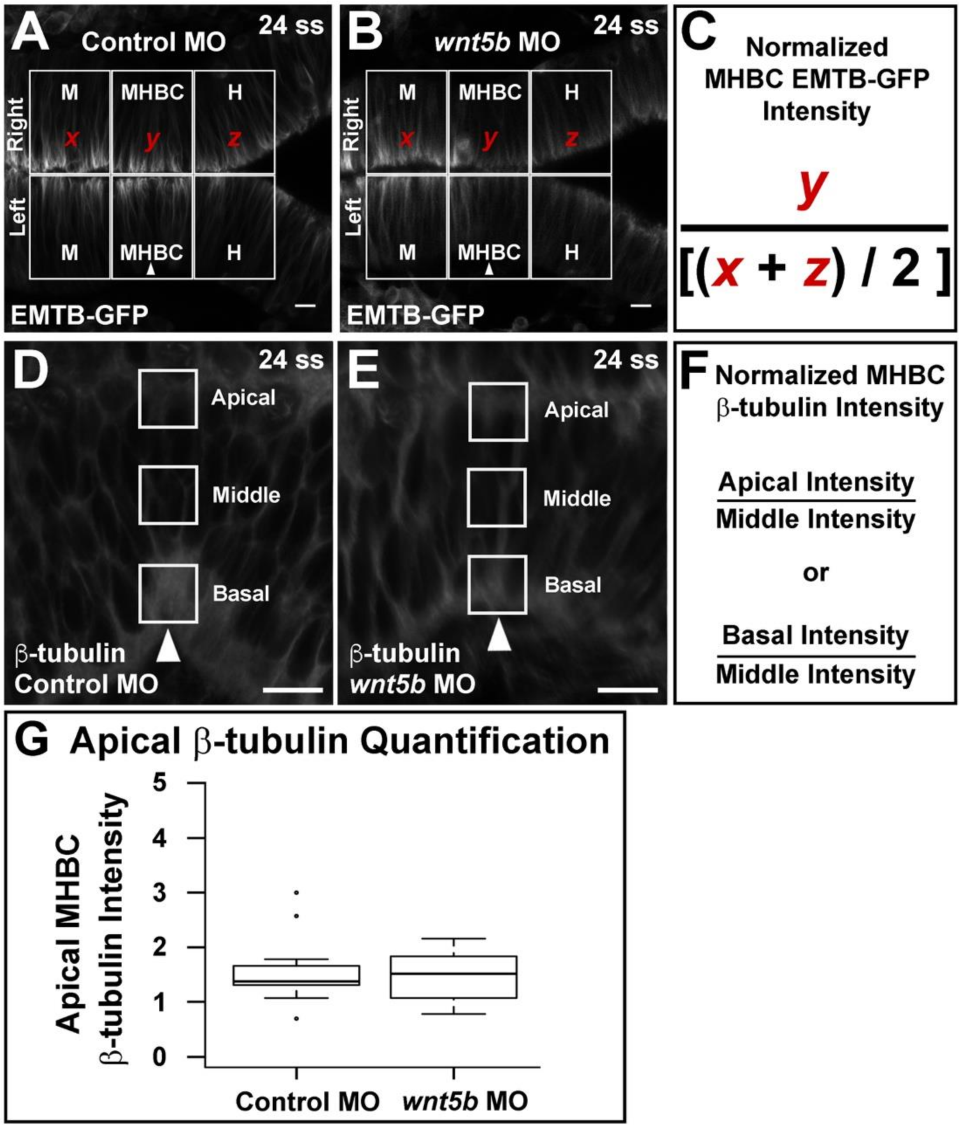

Fig. S4. Quantification of microtubules using live imaging of EMTB-GFP and β-tubulin immunohistochemistry. (A,B) Representative images of a 10 µm Z-series average intensity projection. Boxed area indicates regions where average EMTB-GFP intensity was quantified. Intensity at the midbrain was averaged with the intensity at the hindbrain. The intensity measured at the MHBC was divided by the averaged intensity of the midbrain and hindbrain, for each embryo side. Left and Right. M, midbrain. MHB, midbrain-hindbrain boundary. H, hindbrain. (C) Formula for normalizing MHBC average intensity per side of neural tube. This normalization was used for comparison of MHBC intensity across embryos and experiments. First, midbrain and hindbrain intensity is averaged. MHB intensity is then divided by the averaged midbrain-hindbrain intensity. Final comparisons are made between Control and *wnt5b* MO injected embryos. (D,E) Representative images of a 10 µm Z-series average intensity projection of β-tubulin immunostaining. Boxed areas indicate where β-tubulin average intensity was quantified for apical, middle, and basal regions of the MHBC within a single embryo. The average intensity at either the apical MHBC region or the basal MHBC region is divided by the average intensity at the middle MHBC region in the same embryo to acquire apical or basal MHBC β-tubulin intensity, respectively. (F) Formula for normalizing MHBC apical or basal

Development • Supplementary information

average intensity. This normalization was used for comparison of basal MHBC intensity across embryos and for comparisons between Control MO and *wnt5b* MO injected embryos. (G) Quantification of the normalized Apical β-tubulin intensity at the MHBC in Control versus *wnt5b* morphants. Apical MHBC intensity was divided by the intensity in the middle of the cell. Box plots indicate the 25th and 75th percentiles and the median. Three independent experiments are represented. Control MO, *n*=7; *wnt5b* MO, *n*=6. Scale bars: 10 μm.

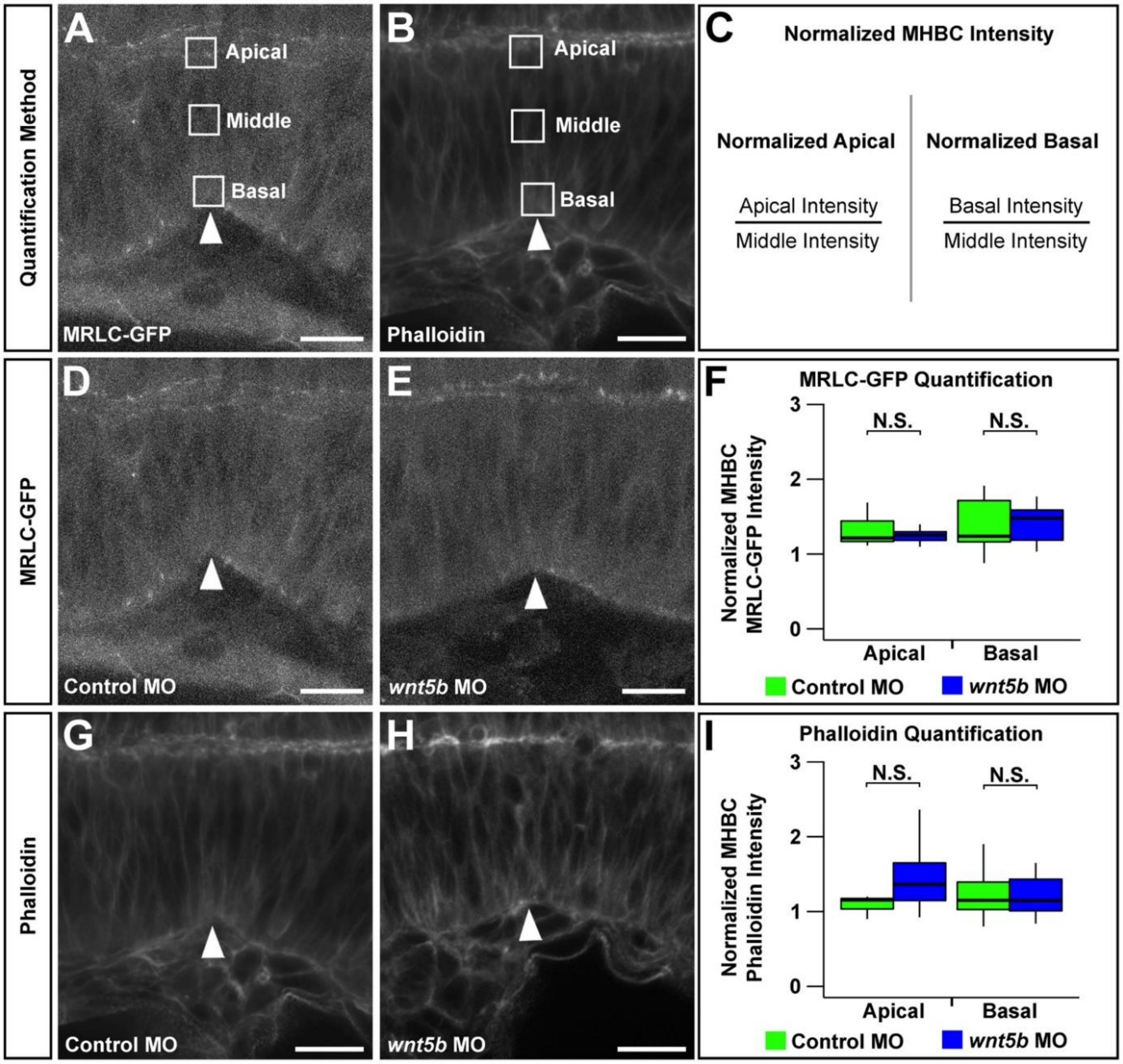

Fig. S5. *wnt5b* knockdown does not affect apical or basal localization of myosin regulatory light chain (MRLC) or actin at the MHBC. To assess the potential role of *wnt5b* in mediating the actomyosin network at the MHBC, we examined MRLC localization using live imaging of GFP-tagged myosin regulatory light chain (MRLC-GFP) and examined actin localization using fixed tissue imaging of AlexaFluor-488 phalloidin-stained embryos. (A,B) Representative confocal image of MRLC-GFP mRNA injected embryo (A) or phalloidin stained embryo (B) showing 10 μm average intensity projections at 24 ss. Boxed areas indicate where average intensity of either MRLC-GFP or phalloidin was quantified for apical, middle, and basal regions of the MHBC cells. (C) Formula is shown for normalizing apical or basal MHBC average intensity. Briefly, either apical or basal MHBC average intensity is divided by the average intensity of the middle MHBC region within the same embryo, for each embryo. This normalization was used for comparison of apical or basal MHBC intensity across embryos and experiments. (D,E) Representative confocal images of 10 μm average intensity projections at 24 ss of wild-type embryos co-injected with 150 pg/embryo MRLC-GFP, 50 pg/embryo memCherry, 3 pg/embryo p53 MO and either 3 pg/embryo Control MO (D) or 3 pg/embryo *wnt5b* MO (E). (F) Quantification of the normalized apical and basal MHBC MRLC-GFP intensity in Control versus

Development • Supplementary information

wnt5b morphants demonstrate no significant difference apically or basally. Box plots indicate the 25th and 75th percentiles and the median. Three independent experiments are represented. Control MO, *n*=9; *wnt5b* MO, *n*=8. Scale bars: 10 µm. (G,H) Representative confocal images of 10 µm average intensity projections at 24 ss of phalloidin stained wild-type embryos that were co-injected with 3 pg/embryo p53 MO and either 3 pg/embryo of Control MO (G) or 3 pg/embryo *wnt5b* MO (H). For phalloidin staining, embryos were fixed at 24 ss in 4% paraformaldehyde in phosphate buffered saline with 0.1% Tween (PBT) for two hours, washed with PBT three times for 10 minutes each, deyolked, and incubated in phalloidin (1:40, A12379, Invitrogen) in PBT overnight at 4 degrees C. Next, embryos were washed three times for ten minutes each in PBT, flat-mounted in glycerol, and imaged using confocal microscopy. (H) Quantification of the normalized apical and basal MHBC intensity in Control versus *wnt5b* morphants demonstrated no significant differences. Box plots indicate the 25th and 75th percentiles and the median. Three independent experiments are represented. Control MO, *n*=6; *wnt5b* MO, *n*=7. N.S. (*not significant*) indicates *P*>0.05. Scale bars: 10 µm.

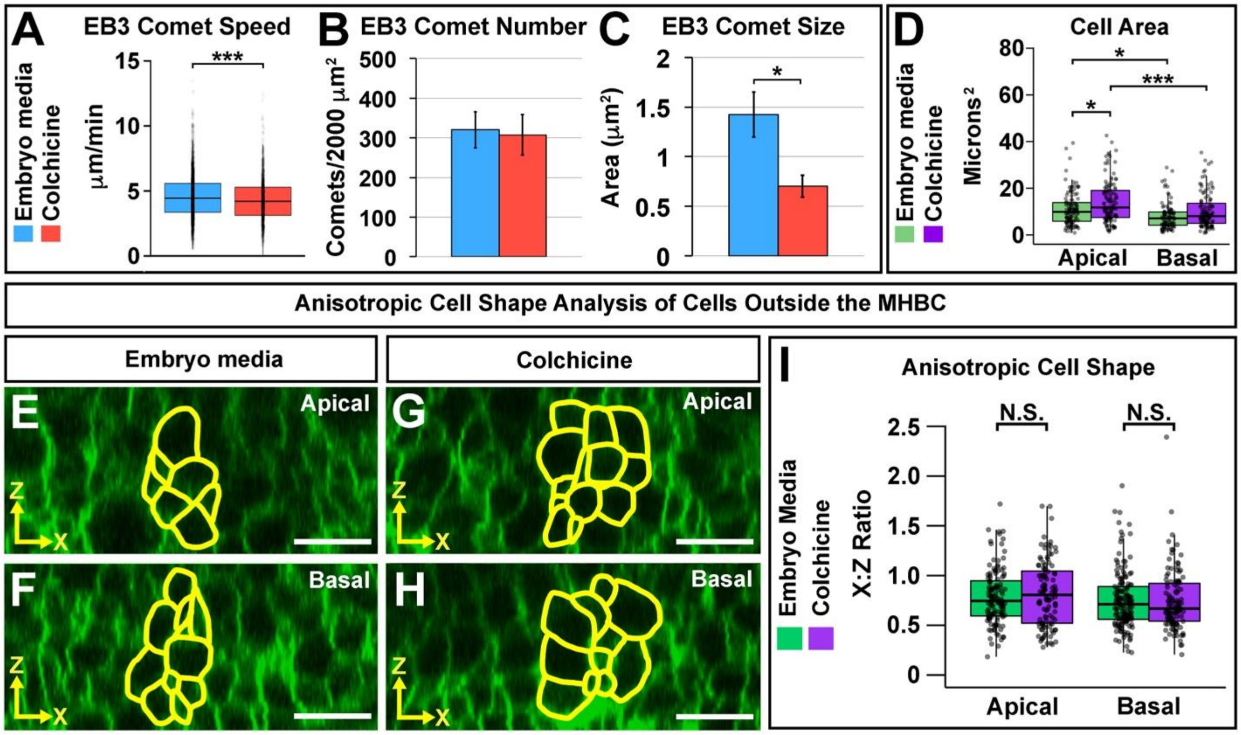

Fig. S6. Colchicine treatment affects microtubule dynamics at the MHBC but does not affect basal MHBC cell area or cell shape outside the MHBC. (A-C) Quantification and comparison of 21-24 ss embryos injected with EB3-GFP mRNA and treated at 18 ss with either embryo media or Colchicine. Microtubule plus-end EB3-GFP comet speed in microns per minute (A), EB3-GFP comet number per 2000 μm^2 (B), and EB3-GFP comet size in μm^2 (C). Welch t-tests were conducted to determine significance, * indicated $P<0.05$, *** indicates $P<0.005$. Boxplots indicate the 25th and 75th percentiles and the median. Three independent experiments are represented. Control embryo media, *n*=6; Colchicine, *n*=6. (D) Quantification of apical and basal cell area of 24 ss wild-type embryos injected with memGFP mRNA and treated at 18 ss with either embryo media or Colchicine. * indicates $P<0.05$, *** indicates $P<0.005$. Boxplots indicate the 25th and 75th percentiles and the median. Three independent experiments are represented. Control embryo media, *n*=7; Colchicine, *n*=8. (E-I) Anisotropic cell shape outside of the MHBC was not different between Embryo media and Colchicine treated embryos. Apical (E,G) and basal (F,H) digital slices of WT 24 ss embryos injected with memGFP and treated with either embryo media (E,G) or Colchicine (F,H). Cells that are 40 microns posterior to the MHBC are outlined in yellow. (K) Quantification of X:Z ratio of cells outside the MHBC. Box plots indicate the 25th and 75th percentiles and the median. Three independent experiments are represented. Embryo media, *n=7*; Colchicine*, n=8*. N.S. (*not significant*) indicates $P>0.05$. Scale bars: 10 μm.

Development • Supplementary information

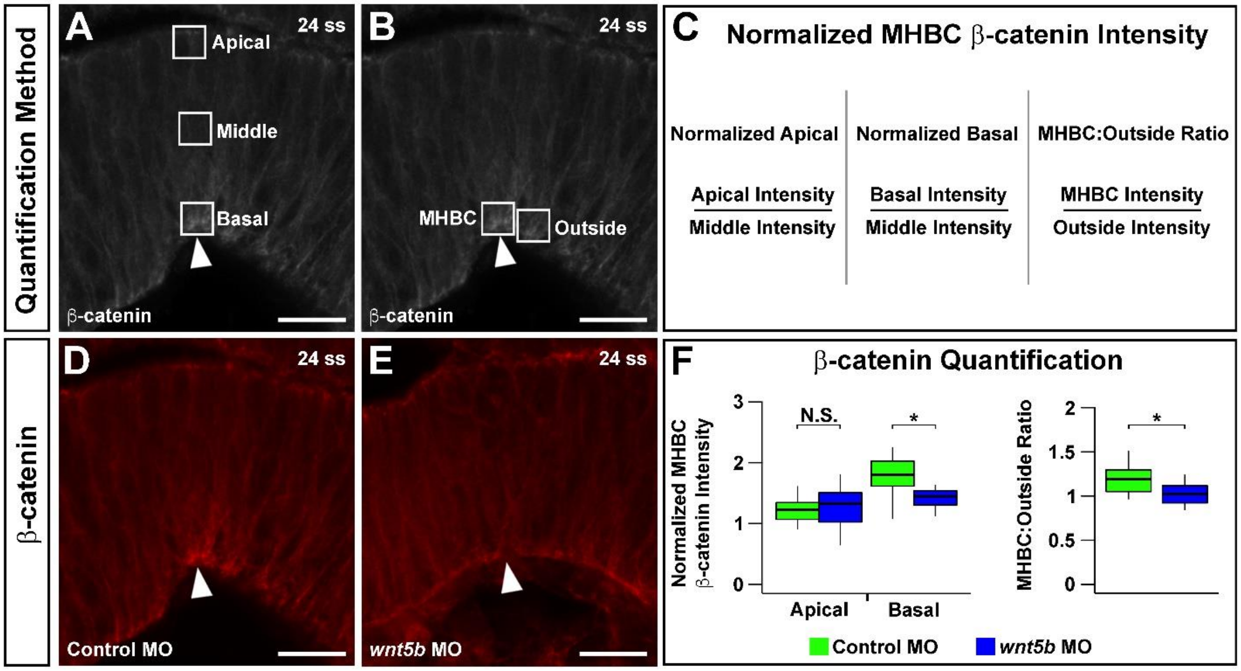

Fig. S7. *wnt5b* knockdown affects basal but not apical localization of β-catenin. In order to examine the effects of *wnt5b* signaling on subcellular variations of the pathway, we examined the localization of β-catenin at the MHBC using immunohistochemistry. Embryos were fixed in Dents (80% Methanol, 20% DMSO) for 1 hour at room temperature and washed in PBT three times for 30 minutes each. Embryos were deyolked, washed three times for 10 minutes in PBT, and incubated in block solution (1% Boehringer Mannheim Blocking Reagent, 10% lamb serum, 80% Maleic Acid Buffer) overnight at 4 degrees C, followed by incubation with 2.5 μg/ml β-catenin primary antibody (ab6301, Abcam) in PBT overnight at 4 degrees C. The next day, embryos were washed four times for 1.5 hrs each in PBT and incubated in secondary antibody overnight at 4 degrees C, 1:2500 goat anti-mouse AlexaFluor-555 (A21422, Invitrogen) in PBT. Next, embryos were washed in PBT three times for 30 minutes each, flat mounted in glycerol, and imaged using confocal microscopy. (A,B) Average intensity projections of a 10 μm Z-series of 24 ss embryos immunostained for β-catenin. Boxed areas indicate where average intensity was quantified for apical, middle, and basal regions of the MHBC and the outside basal region posterior to the MHBC. (C) Formula for normalizing MHBC apical and basal average intensity. Apical or basal MHBC average intensity is divided by the average intensity of the middle MHBC region within the same embryo. MHBC:Outside ratios are acquired by dividing the basal MHBC intensity by the average intensity directly adjacent and posterior to the MHBC region. Control and *wnt5b* MO injected embryos were compared for each normalized intensity. (D,E) Average intensity projection of 10 μm Z-series of 24 ss wild-type embryos co-injected with 3 pg/embryo p53 MO and either 3 pg/embryo Control MO (D) or 3 pg/embryo *wnt5b* MO (E) and immunostained for β-catenin. (F) Quantification of the normalized apical and basal MHBC intensity and MHBC:Outside intensity ratio in Control versus *wnt5b* morphants. Box plots indicate the 25th and 75th percentiles and the median. Three independent experiments are represented. Control MO, *n*=8; *wnt5b* MO, *n*=8. N.S. (*not significant*) indicates P>0.05, * indicates P<0.05. Scale bars: 10 μm.

Development • Supplementary information

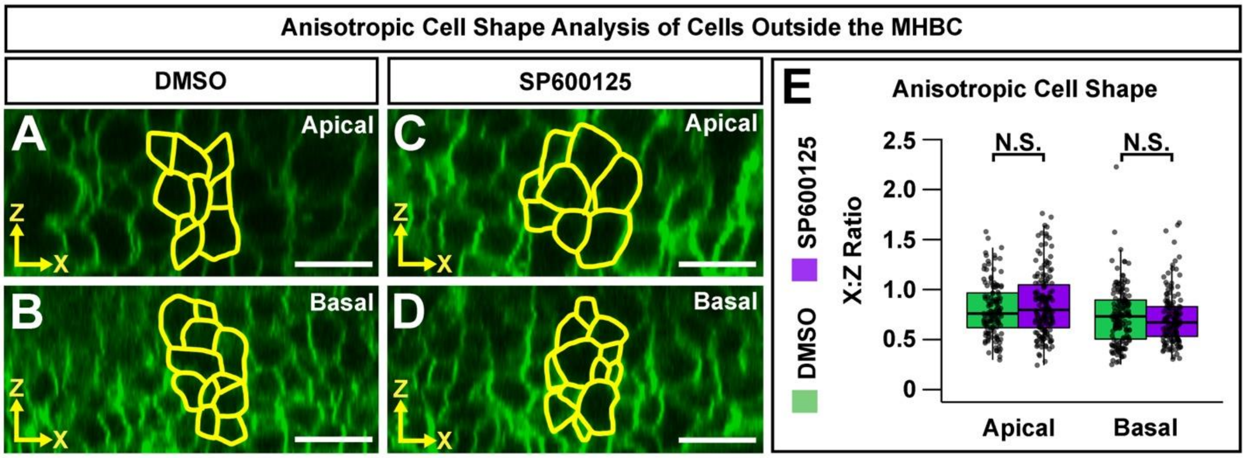

Fig. S8. JNK inhibition does not affect anisotropic cell shape outside the MHBC. (A-D) Apical (A,C) and basal (B,D) digital slices of WT 24 ss embryos injected with memGFP and treated with either DMSO (E,G) or SP600125 (F,H). Cells that are 40 microns posterior to the MHBC are outlined in yellow. (K) Quantification of X:Z ratio of cells outside the MHBC. Box plots indicate the 25th and 75th percentiles and the median. Three independent experiments are represented. DMSO, *n=6*; SP600125*, n=6*. N.S. (*not significant*) indicates *P*>0.05. Scale bars: 10 µm.

Development • Supplementary information

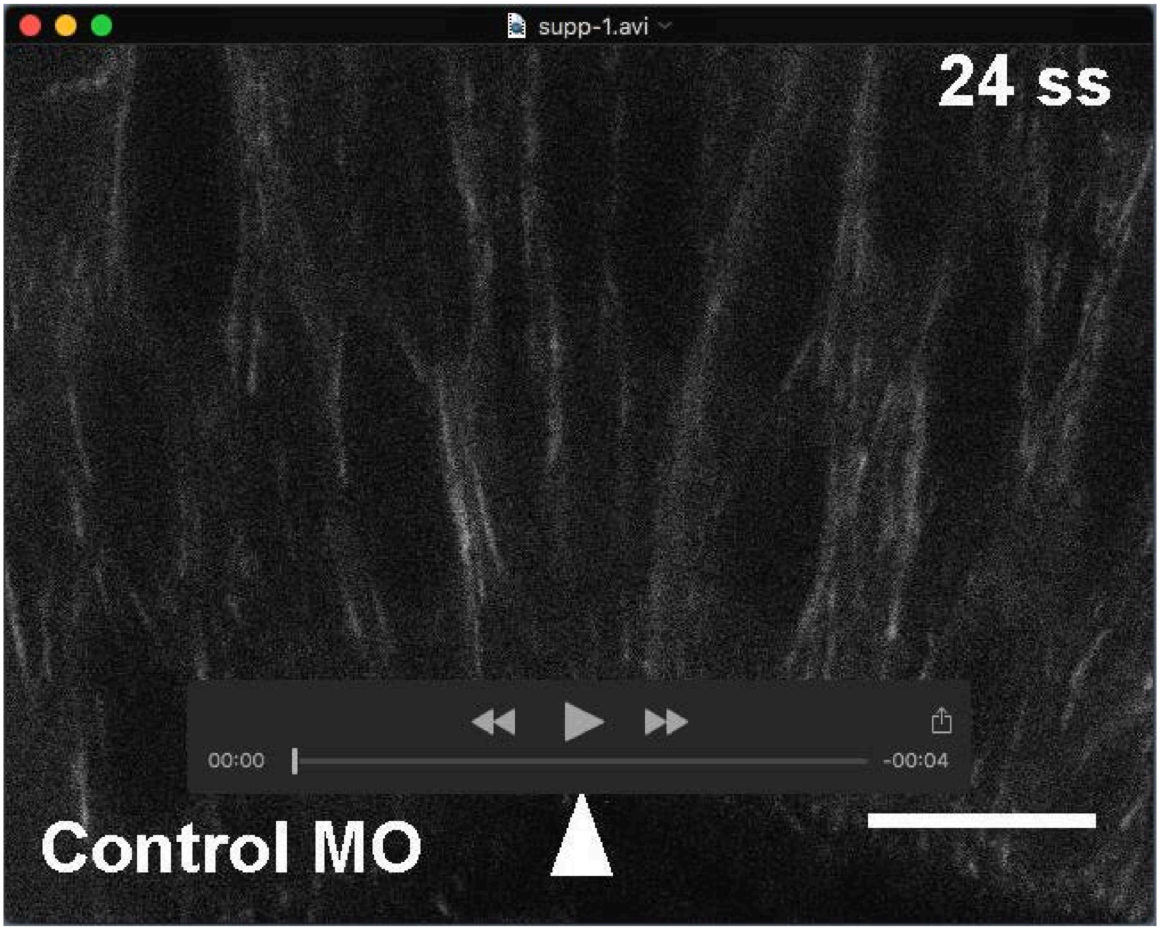

Movie 1. Control MO EB3-GFP Raw Data Timelapse, related to Figure 3. Representative live confocal timelapse at the MHB of an embryo co-injected with memCherry, EB3-GFP mRNA, and Control MO. Timelapse data were acquired at 21-24 ss for 10 minutes at 1 frame per 4 seconds and cropped to 100 seconds. Video plays at 5 frames per second for 5 seconds and shows raw EB3-GFP comets. Arrowhead indicates MHBC. Scale bars: 10 μm.

Development • Supplementary information

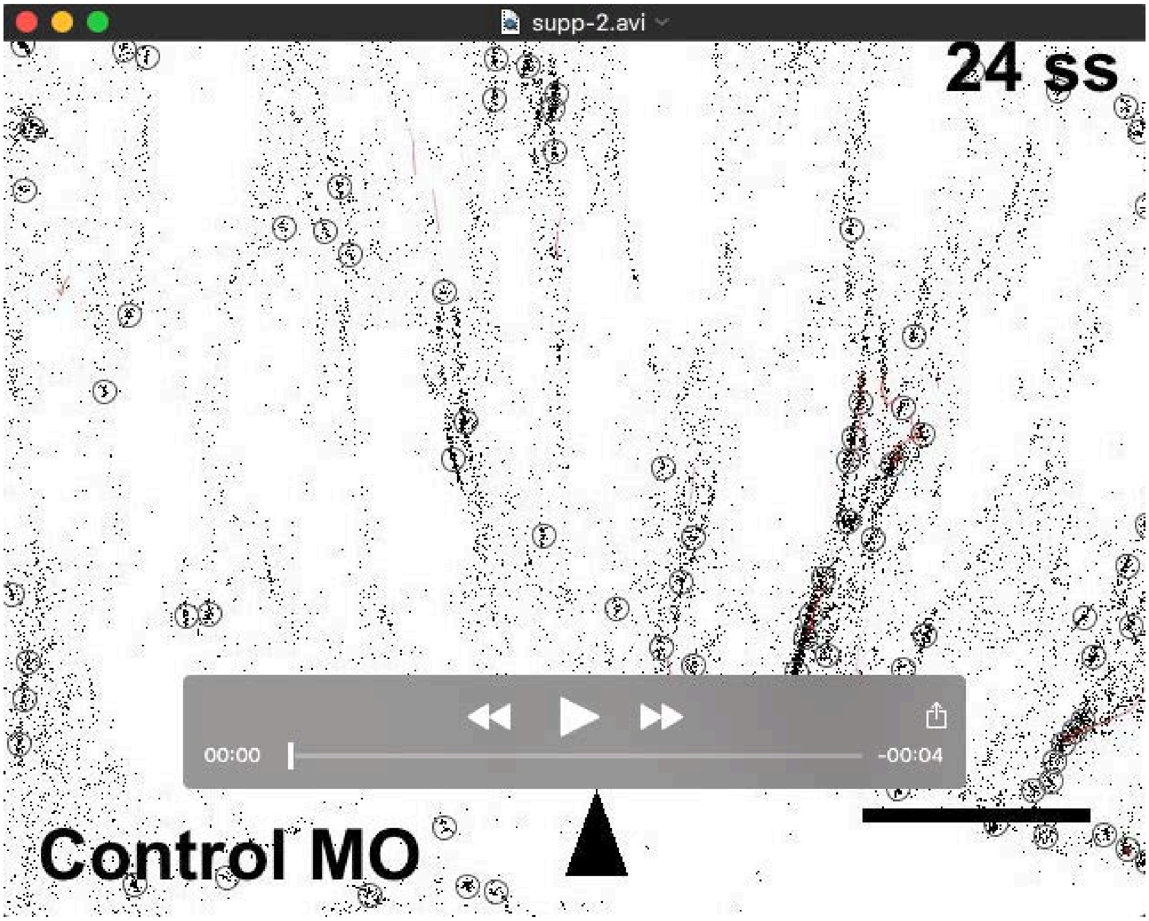

Movie 2. Control MO EB3-GFP Tracks Timelapse, related to Figure 3. Representative live confocal timelapse at the MHB of an embryo co-injected with memCherry, EB3-GFP mRNA, and Control MO. Timelapse data were acquired at 21-24 ss for 10 minutes at 1 frame per 4 seconds and cropped to 100 seconds. Video plays at 5 frames per second for 5 seconds and shows processed EB3-GFP comets and tracks. Images have been processed using the OTSU thresholding method and FIJI Trackmate plugin for particle tracks. Arrowhead indicates MHBC. Scale bars: 10 µm.

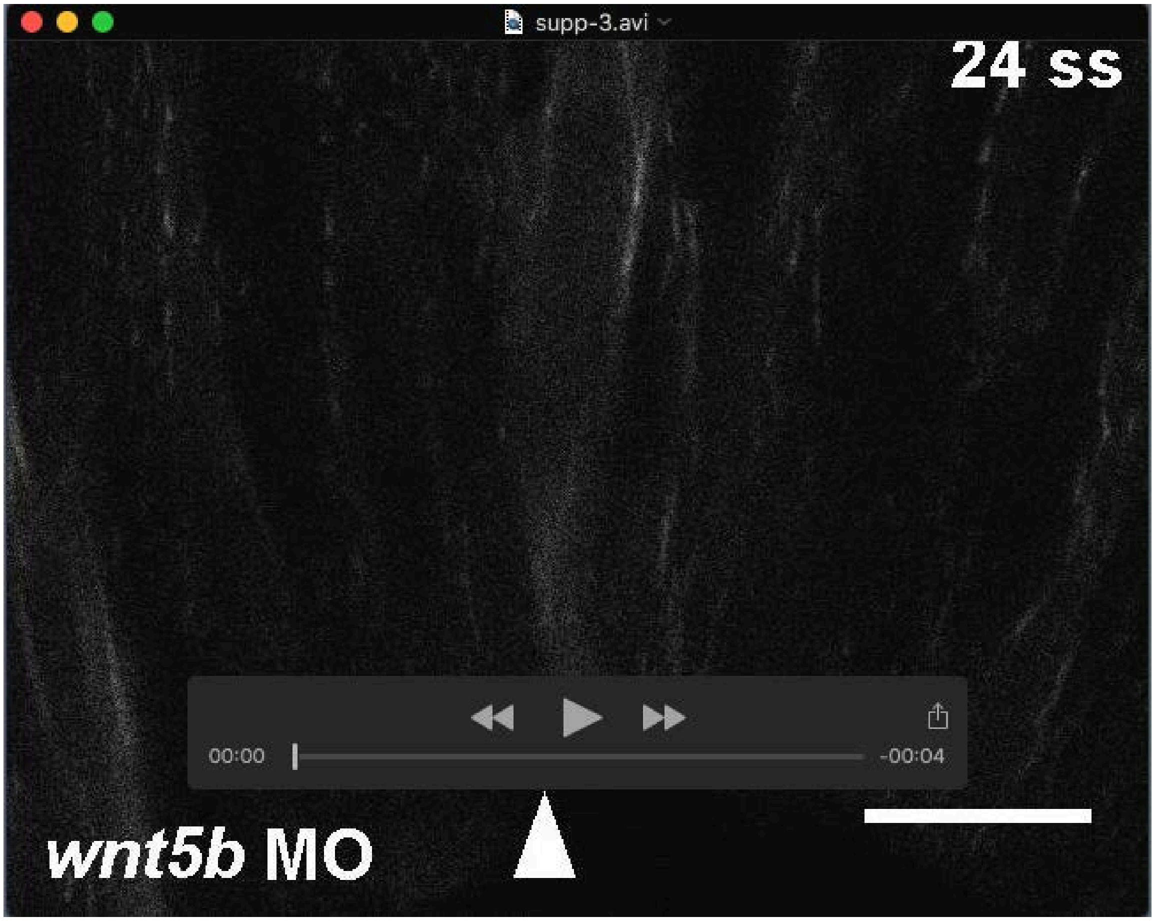

Movie 3. *wnt5b* MO EB3-GFP Raw Data Timelapse, related to Figure 3. Representative live confocal timelapse at the MHB of an embryo co-injected with memCherry, EB3-GFP mRNA, and *wnt5b* MO. Timelapse data were acquired at 21-24 ss for 10 minutes at 1 frame per 4 seconds and cropped to 100 seconds. Video plays at 5 frames per second for 5 seconds and shows raw EB3-GFP comets. Arrowhead indicates MHBC. Scale bars: 10 μm.

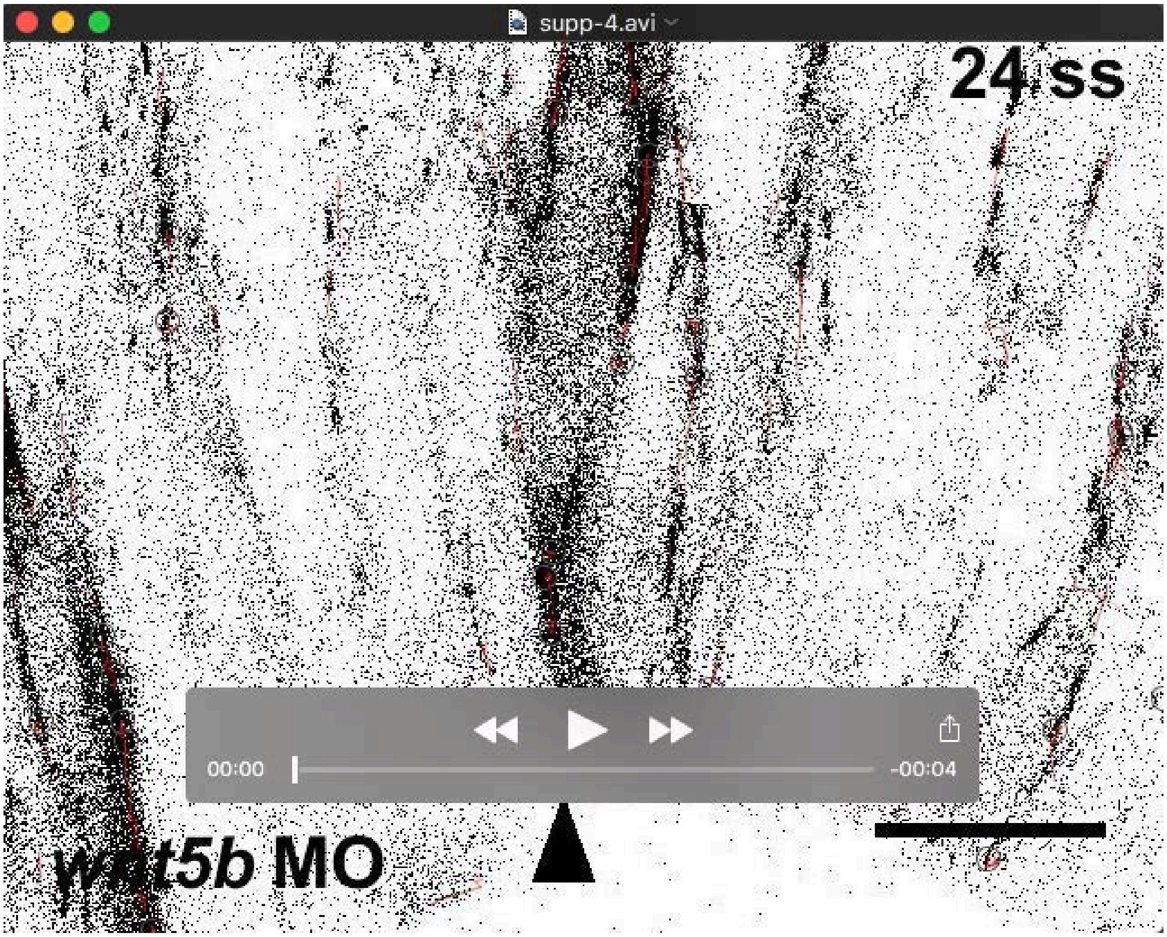

Movie 4. *wnt5b* MO EB3-GFP Tracks Timelapse, related to Figure 3. Representative live confocal timelapse at the MHB of an embryo co-injected with memCherry, EB3-GFP mRNA, and *wnt5b* MO. Timelapse data were acquired at 21-24 ss for 10 minutes at 1 frame per 4 seconds and cropped to 100 seconds. Video plays at 5 frames per second for 5 seconds and shows processed EB3-GFP comets and tracks. Images have been processed using the OTSU thresholding method and FIJI Trackmate plugin for particle tracks. Arrowhead indicates MHBC. Scale bars: 10 μm.

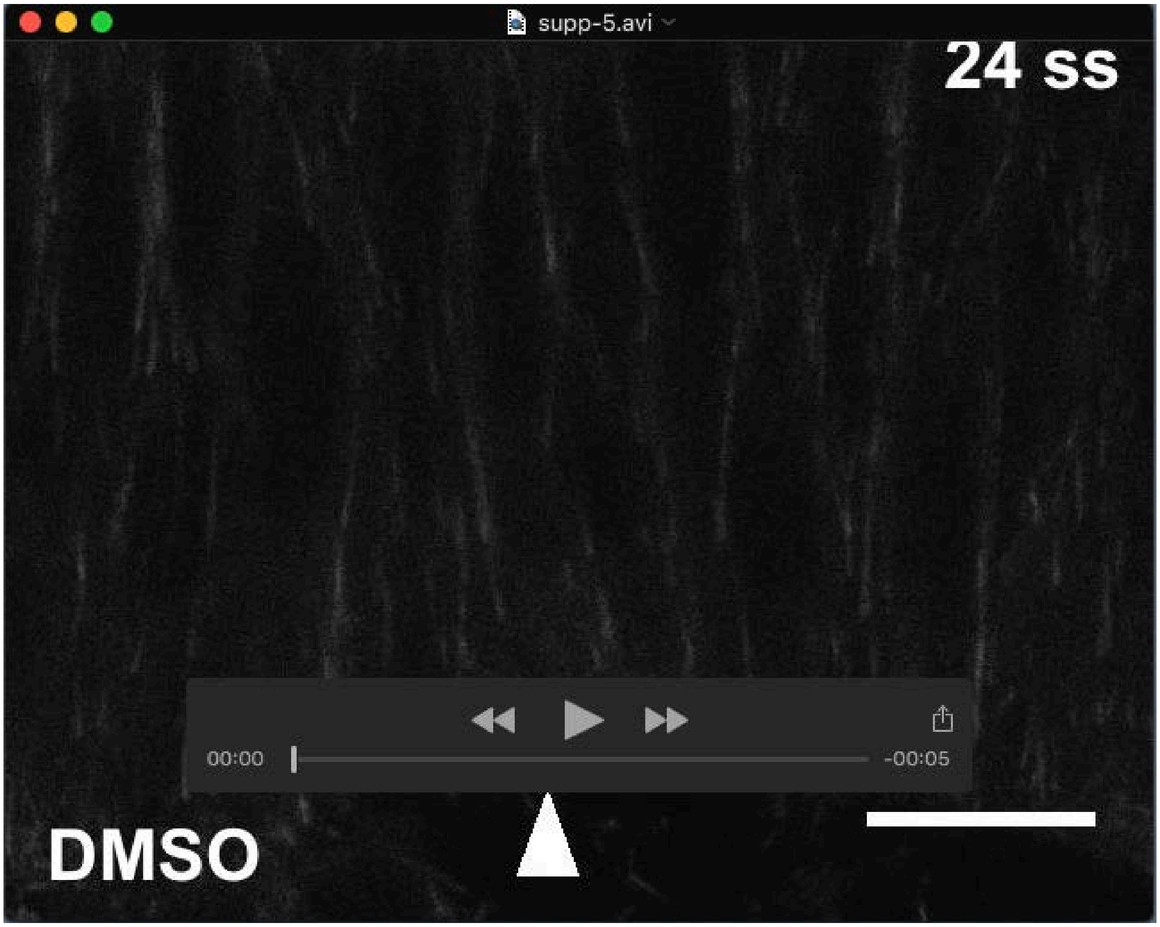

Movie 5. DMSO EB3-GFP Raw Data Timelapse, related to Figure 6. Representative live confocal timelapse at the MHB of an embryo co-injected with memCherry, EB3-GFP mRNA, and treated at 18 ss with DMSO. Timelapse data were acquired at 21-24 ss for 10 minutes at 1 frame per 4 seconds and cropped to 100 seconds. Video plays at 5 frames per second for 5 seconds and shows raw EB3-GFP comets. Arrowhead indicates MHBC. Scale bars: 10 μm.

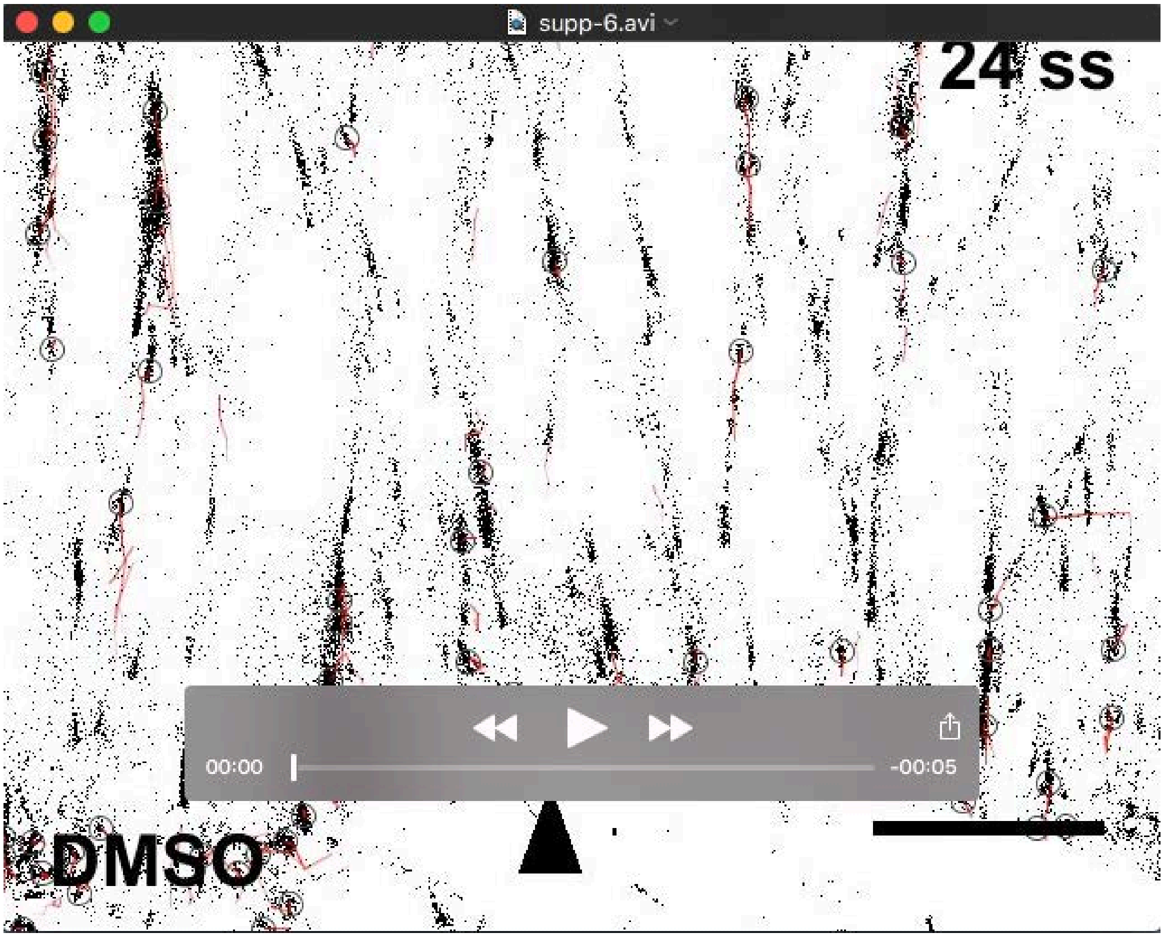

Movie 6. DMSO EB3-GFP Tracks Timelapse, related to Figure 6. Representative live confocal timelapse at the MHB of an embryo co-injected with memCherry, EB3-GFP mRNA, and treated at 18 ss with DMSO. Timelapse data were acquired at 21-24 ss for 10 minutes at 1 frame per 4 seconds and cropped to 100 seconds. Video plays at 5 frames per second for 5 seconds and shows processed EB3-GFP comets and tracks. Images have been processed using the OTSU thresholding method and FIJI Trackmate plugin for particle tracks. Arrowhead indicates MHBC. Scale bars: 10 μm.

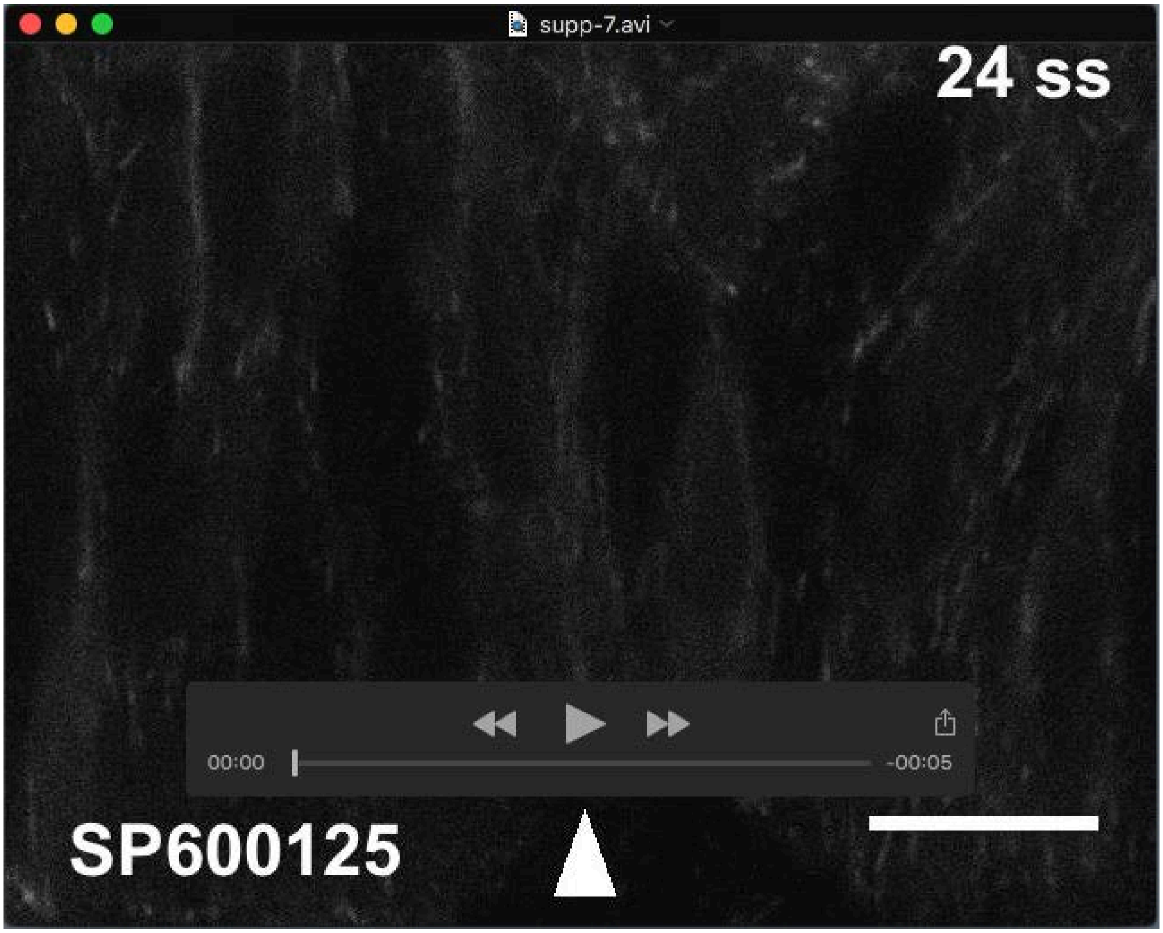

Movie 7. SP600125 EB3-GFP Raw Data Timelapse, related to Figure 6. Representative live confocal timelapse at the MHB of an embryo co-injected with memCherry, EB3-GFP mRNA, and treated at 18 ss with SP600125. Timelapse data were acquired at 21-24 ss for 10 minutes at 1 frame per 4 seconds and cropped to 100 seconds. Video plays at 5 frames per second for 5 seconds and shows raw EB3-GFP comets. Arrowhead indicates MHBC. Scale bars: 10 μm.

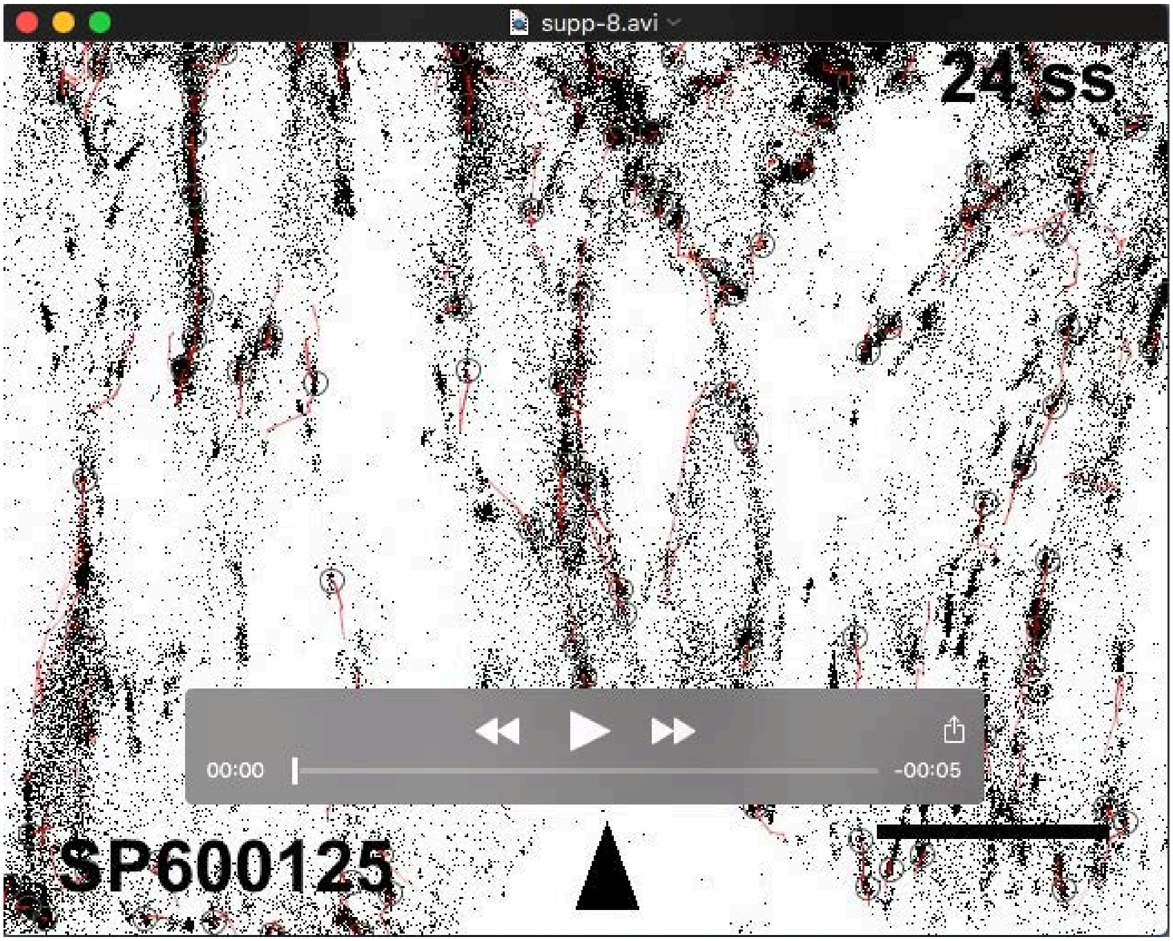

Movie 8. SP600125 EB3-GFP Tracks Timelapse, related to Figure 6. Representative live confocal timelapse at the MHB of an embryo co-injected with memCherry, EB3-GFP mRNA, and treated at 18 ss with SP600125. Timelapse data were acquired at 21-24 ss for 10 minutes at 1 frame per 4 seconds and cropped to 100 seconds. Video plays at 5 frames per second for 5 seconds and shows processed EB3-GFP comets and tracks. Images have been processed using the OTSU thresholding method and FIJI Trackmate plugin for particle tracks. Arrowhead indicates MHBC. Scale bars: 10 µm.
